# Equine skeletal muscle adaptations to exercise and training: evidence of differential regulation of autophagosomal and mitochondrial components

**DOI:** 10.1186/s12864-017-4007-9

**Published:** 2017-08-09

**Authors:** Kenneth Bryan, Beatrice A. McGivney, Gabriella Farries, Paul A. McGettigan, Charlotte L. McGivney, Katie F. Gough, David E. MacHugh, Lisa M. Katz, Emmeline W. Hill

**Affiliations:** 1 UCD School of Agriculture and Food Science, University College Dublin, Belfield, D04 V1W8 Ireland; 2 UCD Conway Institute of Biomolecular and Biomedical Research, University College Dublin, Belfield, D04 V1W8 Ireland; 3 UCD School of Veterinary Medicine, University College Dublin, Belfield, D04 V1W8 Ireland

**Keywords:** Equine, Skeletal muscle, Transcriptome, Exercise, Training, RNAseq, Functional module, Network analysis, Mitochondria, Autophagy

## Abstract

**Background:**

A single bout of exercise induces changes in gene expression in skeletal muscle. Regular exercise results in an adaptive response involving changes in muscle architecture and biochemistry, and is an effective way to manage and prevent common human diseases such as obesity, cardiovascular disorders and type II diabetes. However, the biomolecular mechanisms underlying such responses still need to be fully elucidated. Here we performed a transcriptome-wide analysis of skeletal muscle tissue in a large cohort of untrained Thoroughbred horses (*n* = 51) before and after a bout of high-intensity exercise and again after an extended period of training. We hypothesized that regular high-intensity exercise training primes the transcriptome for the demands of high-intensity exercise.

**Results:**

An extensive set of genes was observed to be significantly differentially regulated in response to a single bout of high-intensity exercise in the untrained cohort (3241 genes) and following multiple bouts of high-intensity exercise training over a six-month period (3405 genes). Approximately one-third of these genes (1025) and several biological processes related to energy metabolism were common to both the exercise and training responses. We then developed a novel network-based computational analysis pipeline to test the hypothesis that these transcriptional changes also influence the contextual molecular interactome and its dynamics in response to exercise and training. The contextual network analysis identified several important hub genes, including the autophagosomal-related gene *GABARAPL1*, and dynamic functional modules, including those enriched for mitochondrial respiratory chain complexes I and V, that were differentially regulated and had their putative interactions ‘re-wired’ in the exercise and/or training responses.

**Conclusion:**

Here we have generated for the first time, a comprehensive set of genes that are differentially expressed in Thoroughbred skeletal muscle in response to both exercise and training. These data indicate that consecutive bouts of high-intensity exercise result in a priming of the skeletal muscle transcriptome for the demands of the next exercise bout. Furthermore, this may also lead to an extensive ‘re-wiring’ of the molecular interactome in both exercise and training and include key genes and functional modules related to autophagy and the mitochondrion.

**Electronic supplementary material:**

The online version of this article (doi:10.1186/s12864-017-4007-9) contains supplementary material, which is available to authorized users.

## Background

Equine athletes have a genetic heritage that has been influenced by millions of years of evolution as grazing animals on prairie and steppe. More recently, centuries of intense selective breeding in the Thoroughbred horse has led to the refinement of multiple physiological adaptations for athletic performance, resulting in an ideal model of a natural athlete for the investigation of exercise and adaptive training responses. Equine skeletal muscle shows a remarkable ability to adapt to physical exercise and long-term training; however, the genetic, epigenetic and molecular changes underlying these adaptive responses have yet to be fully elucidated [1–3]. Cyclic muscle contraction during repeated bouts of exercise over time (training or conditioning) is known to induce physiological adaptation in skeletal muscle, which exhibits remarkable plasticity in structure and function [4]. In equine athletes, training generally leads to an increase in muscle mass and aerobic capacity but the specific response, in terms of muscle fibre type and metabolic adaptation, depends mainly on the type of training regime (e.g. endurance or sprint type exercise) [5–7], nutrition [8–10] and an individual’s specific genetic potential [11–13]. Principal genetic determinants of muscle fibre type include the myostatin gene (*MSTN*), which encodes a ligand of the TGF-beta receptor family that negatively regulates muscle growth, and that has been associated with strength and performance in both human [14] and equine athletes [15]. Sequence polymorphisms in the equine *MSTN* gene are highly predictive of optimal race distance in Thoroughbred horses [13, 15–19]. Horses homozygous for the ‘sprint’ variant (C-allele or SINE insertion) have 12.5% more type *2X* myofibers than horses with the alternate allele [20]. Following a period of training in Thoroughbred horses an increase in type *2A* and a concurrent decrease in type *2X* fibres along with an overall increase in muscle mass is typically observed [6]. As type *2A* fibres can sustain high power outputs for longer than *2X* the functional implication of this is increased endurance. Concurrent with changes in muscle mass and fibre type, exercise training elicits metabolic adaptations. This primarily involves an increased capacity for oxidative phosphorylation [5], increased mitochondrial density [21] and a shift toward oxidizing proportionately more fats and less glucose during exercise [22].

It has been hypothesized that the adaptive response to training is caused by incremental changes in gene expression following a single bout of exercise, which will “accumulate” during the traing period leading to new baseline levels of gene expression. This would result in a significant overlap in the exercise and training response genes [23–25]. The alternate hypothesis is that transient differential expression of genes in response to exercise precedes adaptive changes through secondary mechanisms. In this case little overlap would be seen between the exercise and training response genes [26]. While there is an assumption that “accumulative” changes play a major role in the adaptive response no study has clearly demonstrated this. In human skeletal muscle the mRNA expression of key transcription factors is transiently induced by exercise training leading to increases in downstream transcriptional and mitochondrial proteins [25]. In equine athletes it has been shown that a single bout of high-intensity exercise consisting of an incremental step-test to fatigue elicits a modulation of the expression of genes involved in metabolism and muscle hypertrophy, signatures of endurance and resistance exercise, respectively [27]. Following a period of training the basal levels of genes related to the mitochondrion, oxidative phosphorylation and fatty acid metabolism have been shown to be significantly upregulated [28], supporting the hypothesis that training may cause a transcriptional reprogramming of the muscle.

A range of approaches has been taken to better understand the molecular adaptations to exercise and training with many factors needing to be considered for appropriate experimental design [6, 22, 28–32]. Generally, the analysis of the impact of an experimental variable (e.g. environment, treatment, mutation, disease etc.) on a cell, tissue or organism results in a list of statistically significant response variables, such as genes. It is common, owing to the modular architecture of biological systems, to then examine this list for statistical over-representation of known functional modules (e.g. pathways or complexes) to assist biological interpretation (often referred to pathway enrichment analysis, or similar). Although valuable, such analysis is confined by the limits of the current knowledge of functional modules which tends to be biased [33] and incomplete [34]. However, less supervised approaches that are informed more by the similarity of entity (i.e. gene) behaviour, are more open to uncovering unknown or context-specific gene relationships [34, 35].

The function of most genes, or gene products (proteins, miRNA, lncRNAs, etc.), can only be carried out in combination with other biomolecules as part of a functional module [36]. Therefore, to fully elucidate the functional relevance of a set of genes we must also model the related set of molecular interactions and their dynamics. There are several methods for direct detection [37, 38] or inferral [39–41] of molecular interactions. Once determined, the set of molecular interactions may be modelled as a network of nodes (genes) and edges (interactions) and interrogated with an extensive toolbox of established network analysis methods [34, 42]. Common metrics include the centrality indices ‘degree’ and ‘betweeness’, which are used to measure the importance of individual network nodes in terms of the local connections and their network wide influence respectively. In the context of biomolecular interaction networks, these ‘hub’ and ‘bottleneck’ nodes tend to be fundamentally important to the behaviour of the network [43] and indeed to the biological process, cell, tissue or organism as a whole [44]. Other higher level node grouping or clustering methods, such as ‘community detection’ [45, 46], allow modelling of the underlying functional modules (e.g. complex or pathway) in a particular molecular interaction network [36, 42]. Other methods extend this concept further to analyse multi-state, or ‘dynamic’ networks recorded over various time points or experimental treatments [47].

Although hundreds of thousands of protein-protein interactions (PPIs) have been recorded across many organisms, cell types and experimental conditions and made publicly available [48], proteome-scale detection of PPIs still remains beyond the budget and expertise of most researchers. Genome-wide gene expression analysis, however, is now relatively affordable and commonplace. Thus, high-throughput gene expression data generated for an experimental context is often integrated with the universal set of known molecular interactions (i.e. the global interactome) to infer putative contextual molecular interaction networks. However, most implementations of this approach only attempt to predict the contextual state of the nodes in the network (e.g. the set of genes expressed in or associated with the condition of interest) and include all associated PPIs as edges, regardless of the experimental conditions or cell types in which they were detected, which is clearly a gross over-simplification [49–51]. It has been recognized for over a decade that genes that exhibit highly correlated co-expression profiles, as determined by Pearson’s correlation coefficient for example, often interact within the same biological module [40, 52]. This raises the prospect of using gene expression information to also infer the contextual state of the edges in a molecular interaction network but this in itself is hampered by exceedingly high false positive rates [41]. Here we have developed a novel pipeline to integrate contextual information, derived from gene expression data, with publicly available PPI data [48] to predict both the nodes and edges in the contextual PPI network. Ultimately, this more refined contextual model should lead to an improved elucidation of the interactions and functional modules that are active in each of the experimental conditions considered in this study.

In summary, the aim of this study was to investigate changes in the transcriptome of the skeletal muscle of untrained Thoroughbred horses (UR) in response to a single bout of high-intensity sprint exercise (UE), and following an exercise conditioning (training) regime (TR). We hypothesised that regular bouts of high-intensity exercise training would prime the transcriptome of Thoroughbred skeletal muscle for the demands of the next exercise bout. Furthermore, by integrating this information on contextual transcriptional changes with known molecular interactions, we also tested the hypothesis that these transcriptional adaptions may lead to a ‘re-wiring’ of the molecular interactome in response to high-intensity exercise and training. Lastly we have demonstrated that a refined computational network-based approach, which considers both context-specific nodes (genes) and edges (interactions), has the potential to uncover novel features indicative of specific biological processes when compared to standard supervised approaches.

## Methods

### Study cohort

University College Dublin Animal Research Ethics Committee approval, a licence from the Department of Health (B100/3525) and explicit owner/trainer informed consent were obtained for the use of all horses and procedures in this study.

The study cohort comprised a subset of Thoroughbred horses, trained for Flat racing at a single training establishment under the management of a single trainer. The UR cohort comprised of yearling Thoroughbred horses (*n* = 51; 23 males and 28 females; 19.5 ± 1.5 months old) in submaximal training prior to entering sprint training. The UE cohort consisted of the same group of horses undertaking their first or second ‘work day’ (WD, high-intensity sprint exercise simulating a race) on an all-weather gallop. The TR cohort consisted of horses at the end of the racing season following approximately six months of sprint training. All horses in this group had achieved “race fitness” with an average of 15.1 ± 9.1 SD WDs (range 6–43). Table 1 summarises details of sampling and physiological measurements.

**Table 1:** Cohort information and experimental conditions

	Untrained Rest	Untrained Exercise	Trained Rest
	(UR)	(UE)	(TR)
*n*	22	17	22
Male	8	8	8
Female	16	9	16
Mean age in days (Range)	590 (513–673)	765 (649–896)	947.6 (884–1051)
Exercise parameters (WD1):			
*-Mean Peak Velocity (m/s)*	.	16.2 (14–17.1)	.
*-Mean Peak Heart Rate (bpm)*	.	224 (212–233)	.
*-Mean Peak Plasma Lactate (mmol/l)*	.	26.7 (20.8–32.2)	.
Mean sub-maximal training weeks prior to sampling (canter)	0	4.7 (1.6–13.1)	27.7 (10.1–47.0)
Mean work days prior to sampling:	0	1	15.1 (6–43)
*n = 0*	22		
*n = 1*	0	17	0
*n > 1*	0	0	22
*> 5 n ≤ 11*	0	0	8
*> 10 n ≤ 20*	0	0	9
*n > 20*	0	0	5

Number of individuals (*n*), males and females, and mean age are given for experimental cohorts Untrained Rest (UR), Untrained Exercise (UE) and Trained Rest (TR). Details of exercise and training regimens for each Thoroughbred cohort prior to sampling day are also shown. Sampling of the Untrained Exercise (UE) cohort was performed ~4 h post-exercise on the first work day (WD1). Sampling of the Trained Rest (TR) cohort was performed after an average of ~15 WDs over a six-month training period

### Tissue sampling time-points

All resting skeletal muscle samples (see protocol below) were collected between 7:30 am and 11:30 am. UE samples were collected from a subset of the UR horses (*n* = 46; 23 males and 23 females; 24.6 ± 2.3 months old) approximately four hours following high-intensity exercise. The study cohort comprised horses in active race training and therefore the number of biopsies that could be sampled was limited. A single 4 h post-exercise time-point was selected that represented the time previously shown to have the greatest number of differentially expressed genes [27] and the greatest magnitude of effect on transcripts in equine skeletal muscle post-exercise [30]. In addition, the time point was selected to avoid potential disruption to routine activity on the yard.

TR samples were collected at rest following a sprint training period of approximately six months from a subset of the UR horses (*n* = 22; eight males, and 14 females; 31.1 ± 1.5 months old). Only horses that were sampled as part of the UR cohort and had a matching sample in the UE or the TR cohort were used in subsequent analysis. The final experimental cohorts contained *n* = 22 (UR), *n* = 17 (UE) and *n* = 22 (TR) horses. Summary statistics are shown in Table 1.

### Exercise and training protocols

Horses exercised six days per week, with gradual introduction and increased frequency of WDs, following which horses entered competitive racing. Training was modified based on soundness, fitness and aptitude, with all decisions made by a single trainer. Prior to exercise, each horse was fitted with a heart rate (HR) telemetry system (Polar Equine S810i heart rate monitor system) and global positioning system (GPS, GPSports Systems SPI10, Canberra, Australia) which recorded speed, HR and exercise distance. The WD exercise protocol was as follows: horses were warmed-up on a horse walker for 10 min (walk and trot) and then walked under saddle for 5–10 min. On the gallop, horses walked for 300 m, trotted for 700 m, walked for approximately 100 m and then galloped up to a maximum velocity for approximately 500-800 m (average distance of 698.8 ± 223.9 m).

### Biopsy sampling and RNA sequencing protocols

Percutaneous needle muscle biopsies (approx. 300 mg) were obtained from the ventral compartment of the middle gluteal muscle from standing unsedated horses using a previously described method [53] and preserved in RNA*later* (Thermo Fisher, Massachusetts, United States). Total RNA was extracted from approximately 70 mg tissue, using a protocol combining TRIzol reagent (Thermo Fisher, Massachusetts, United States), DNase treatment (RNase free DNase) (Qiagen, Hilden, Germany) and RNeasy Mini-Kit (Qiagen). RNA was quantified using a Nano Drop ND1000 spectrophotometer V 3.5.27 and RNA quality and purity were assessed using the 18S/28S ratio and RNA integrity number (RIN) on an Agilent Bioanalyser with the RNA 6000 Nano LabChip kit6 (Agilent, Cork, Ireland). The RNA isolated from UR, UE and TR samples had an average RIN of 8.1 (7.2–9.3), 8.2 (7–9.1) and 8.1 (5.4–8.6), respectively. RNA sequencing was performed by the Research Technology Support Facility Michigan State University. Indexed, strand-specific Illumina sequencing libraries were prepared using the TruSeq Stranded mRNA Library Preparation Kit LT (Illumina, San Diego, United States). Libraries were pooled with ten indexed libraries per pool and sequenced on an Illumina HiSeq 2500 using Rapid Run flow cell and reagents (Illumina). Dual lane loading was employed, meaning a single pool was loaded across both lanes of the flow cell. Each pool was sequenced on one flow cell (two lanes). Sequencing was performed in a 2 × 100 bp paired end (PE100) format. Sequence data was demultiplexed and converted to FastQ format files.

### RNA-seq data pre-processing

*FASTQC* [54] was used to assess sequencing quality and *STAR* [55] was used to align reads to the horse reference genome (Ensembl release 62). *SAMtools* [56] was used to convert files from Sam to sorted indexed Bam format and *featureCounts* [57] was used to assign reads to exons. The correlation between lanes (two technical replicates) for each sample was checked to ensure a correlation of >99% prior to merging data. Differentially expressed (DE) genes were identified using the *limma* [58] R package. Count data was scale normalised using the *calcNormFactors* function from the *EdgeR* [59] package. The voom transformation was applied to the normalised data and a linear model was fitted to the data using the horse as a blocking factor to take into account the pair effect. The empirical Bayes method was used to calculate *P* values. Ensembl IDs of small RNAs (BioMart biotypes: *rRNA, snRNA, misc_RNA, snoRNA, miRNA*) were first removed from the dataset. Differentially expressed (DE) genes were defined as those that were significantly upregulated or downregulated (Benjamini-Hochberg corrected *P*-value <0.05) and that were upregulated or downregulated by at least 25% between the two cohorts being compared (i.e. UE versus UR, TR versus UR).

### Statistical over-representation analysis

To facilitate a complete downstream analysis pipeline, equine Ensembl IDs were first uniquely mapped (one-to-one relationship only) to their better annotated human orthologs, retrieved from the BioMart database [60]. All subsequent analyses were performed with these human orthologs and their annotations. Over-representation of the gene ontology (GO) categories [61] and KEGG and Reactome pathway annotations [62, 63] was performed in an automated manner using the *RDAVIDWebService* [64]. The expected or *background* gene list used in the over-representation analysis included all genes that had a one-to-one mapping with human orthologs and were expressed in skeletal muscle during at least one time point (*n =* 14,688). The statistical significance of gene category over-representation was calculated based on the DAVID software EASE score (*P*-value based on a modified Fisher Exact t-test) [65] and corrected for multiple testing using the Benjamini-Hochberg step-down correction.

### Construction of putative contextual molecular interaction networks

Two dynamic gene interaction networks were constructed, the first to model the molecular interactions before and after exercise in untrained horses (UR vs. UE) and the second to model the training response (UR vs. TR). In each case the set of nodes (genes) in the dynamic network were confined to those that had been found to be differentially expressed in the respective response. An edge (gene interaction) was drawn between nodes (genes) *a* and *b* if (i) there was any previous experimental evidence of a molecular interaction and (ii) if their expression levels were found to be significantly correlated in the respective cohort (i.e. UR, UE or TR). Experimentally validated protein-protein interactions (PPIs) for human, that were annotated to the IMEx (International Molecular Exchange consortium) standard [66], were then obtained (12–09-2016) from the *IntAct* database [48] using the *PSICQUIC* web-service [67] (see Additional file 1 for the specific query used). These interactions were recorded across many cell types, tissues and experimental conditions and represent the global set of all possible interactions in human as defined by *IntAct* DB. Gene expression correlations were calculated across all samples within the relevant cohort (i.e. UR, UE or TR) using Spearman’s rank correlation and only positive, statistically significant correlations (i.e. greater than the minimum critical value given the number of samples in the cohort) were used to generate contextual edges.

### Network analysis

Network analysis and visualization was implemented in the R statistical programming language and primarily used tools from the *igraph* package [68]. The most ‘influential’ nodes (genes) in each network were determined based on both degree (edge number) and betweeness (number of shortest paths that pass through that node) [69]. These metrics highlight the nodes (genes) that have the most influence on information flow in the network at a local (i.e. hubs) and global (i.e. bottlenecks) level respectively. Hubs are scored based simply on node *degree* (number of edges attached to a node) and bottlenecks are scored based on the node *betweeness* (number of shortest paths that pass through a node). The modular or systems level architecture of a network was interrogated in an unsupervised manner (i.e. based only on network topology) by partitioning the nodes (genes) into groups such that the edge (gene interaction) density was higher within groups than between. This was performed using the *fastgreedy* community detection method, which also utilizes edge weights (based on expression correlation in this case) [45] and was also implemented via *igraph*. In practice, we retained only non-trivial clusters (i.e. containing two or more genes) and validated these clusters (putative functional modules) by functional enrichment analysis based on pathways and GO terms (implemented as before).

## Results

### Identification of genes and known functional categories associated with the exercise and training responses

We applied both a fold-change and multiple test correction cut-off to identify differentially expressed genes in the exercise (UR vs. UE) and training (UR vs. TR) responses. We identified 3241 genes that were significantly (BH-corrected *P*-value < 0.05) differentially expressed (>1.25-fold) and mapped uniquely to human orthologs in the exercise response (Additional file 2: Table S1). A similar number of genes (3405) were associated with the training response (Additional file 2: Table S2). Approximately one third of the exercise response genes (1025) were also associated with the training response and 95% of genes were expressed in the same direction (relative to UR) in both responses. There were 2216 and 2380 genes uniquely associated with the exercise or training response respectively.

To attempt to uncover the underlying biological processes involved in the adaptation of skeletal muscle to exercise and training, the gene lists derived from differentially expressed transcripts were assessed for statistical over-representation of known functional modules based on five functional annotation schemas. Two schemas related to biological pathways (curated by KEGG [63] and Reactome [62] databases) and three related to *Cellular Component* (CC), *Molecular Function* (MF) and *Biological Process* (BP) gene ontology (GO) sub-categories [61].

For the exercise response gene list several KEGG and Reactome pathways were found to be significantly over-represented and were observed to fall into one of three general themes, namely: energy metabolism (*Integration of energy metabolism: P =* 4.97 × 10^−11^ *, Diabetes pathways: P =* 3.57 × 10^−06^, *Oxidative phosphorylation: P =* 0.0047 and *Pyruvate Metabolism and TCA Cycle: P =* 0.0204); muscle contraction (*Muscle contraction: P =* 3.57 × 10^−06^ *, Cardiac muscle contraction: P =* 0.0222) and hemostasis (*Hemostasis: P =* 3.76 × 10^−05^ *, Signaling by PDGF: P =* 0.0167). There was also significant enrichment for several pathways related to neurodegenerative diseases (*Alzheimer’s Disease: P =* 4.95 × 10^−06^ *, Huntington’s Disease:* 9.15 × 10^−06^ *and Parkinson’s Disease:* 2.75 × 10^−05^). Statistical enrichment was also observed for similar themes for the GO *Biological Process* sub-category (e.g. *Cellular respiration: P =* 0.0004*, Muscle contraction: P =* 0.0004*, Vasculature development: P =* 0.0307). GO term enrichment also provided more refined annotation relating the *Cellular Component* sub-category (e.g. *Mitochondrion: P =* 4.14 × 10^−15^ *, Contractile fibre: P = 6*.40 × 10^−11^ *, Mitochondrial respiratory chain: P =* 7.41 × 10^−05^) and the *Molecular Function* sub-category (e.g. *Cytoskeletal protein binding: P =* 1.41 × 10^−06^ *, Actin binding: P =* 0.0006*).*

**Fig. 1:**
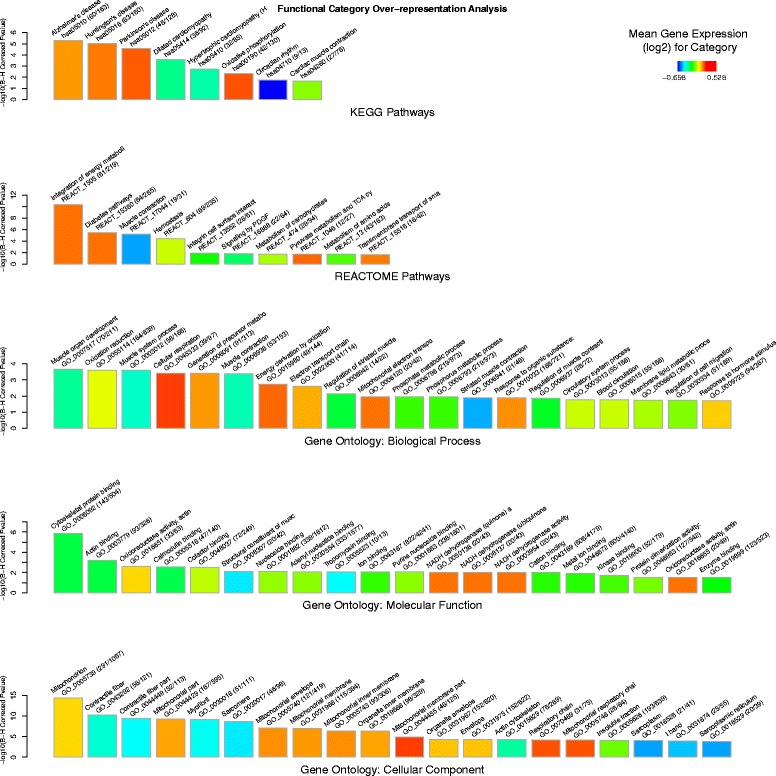
Cellular functions of the exercise response. Bar charts showing over-representation of functional categories (described by KEGG and REACTOME pathways and Gene Ontology: *Biological Process*, *Molecular Function* and *Cellular Component*) for the list of genes (*n* = 3241) that showed statistically significant differential expression, of at least +/− 1.25-fold, between the muscle tissue (*gluteus medius*) from the untrained rest (UR) and the untrained exercise (UE) Thoroughbred cohorts. Bars represent the most significant functional modules (up to 20) for each of the five annotation schemas. Bar height represents statistical significance (*−log*_*10*_ transformed Benjamini-Hochberg (B-H) Corrected *P*-value) of the over-representation, based on the EASE-score (conservative Fisher Exact t-test). Bar color represents the mean differential expression (log_2_(UE/UR) for the genes in this module (see color key). Category name, ID and size (number category genes in gene list/category size) are given above each bar. For example it can be seen that the fourth most significant Gene Ontology:*Biological Process* is ‘Cellular respiration’ and that it is one of the most up-regulated functional categories on average (red color) and that 39 out of the 97 genes assigned to this category are differentially expressed between UE and UR cohorts. Full results are provided in Additional file 2: Table S3

The functional modules related to muscle contraction were the most downregulated modules associated with the exercise response gene list. For example, the largest over-represented module, *Contractile fibre,* contained 56 genes, which were mostly (44/56) downregulated (mean: −1.52-fold) in exercise relative to the untrained rest cohort. These muscle contraction related modules were also found to be unique to the exercise response (i.e. this theme was not found to be associated with the training response, see below). The top twenty most significantly over-represented functional modules in the exercise response gene list are provided in Additional file 2: Table S3 and illustrated via five barcharts (one per annotation schema) in Fig. 1.

**Fig. 2:**
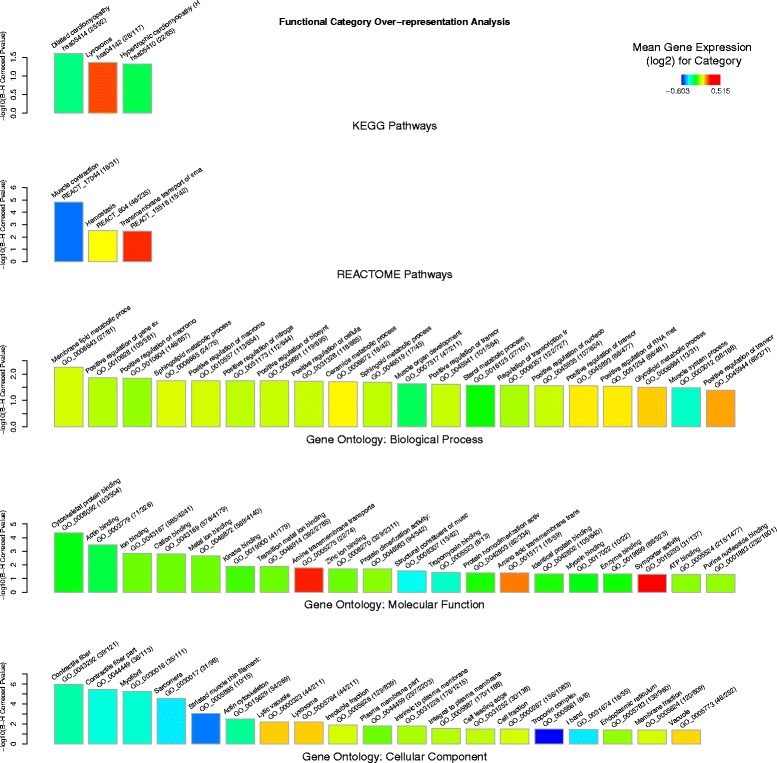
Cellular functions of the exercise-specific response. Bar charts showing over-representation of functional categories (described by KEGG and REACTOME pathways and Gene Ontology: *Biological Process*, *Molecular Function* and *Cellular Component*) for the list of genes (*n* = 2216) that showed statistically significant differential expression, of at least +/− 1.25-fold, between the muscle tissue (*gluteus medius*) from the untrained rest (UR) and the untrained exercise (UE) but not the trained rest (TR) Thoroughbred cohorts. Bars represent the most significant functional modules (up to 20) for each of the five annotation schemas. Bar height represents statistical significance (*−log*_*10*_ transformed Benjamini-Hochberg (B-H) Corrected *P*-value) of the over-representation, based on the EASE-score (conservative Fisher Exact t-test). Bar color represents the mean differential expression (log_2_(UE/UR) for the genes in this module (see color key). Category name, ID and size (number category genes in gene list/category size) are given above each bar. For example it can be seen that the most significant Reactome pathway is ‘Muscle contraction’ and that it is one of the most down-regulated functional categories on average (blue color) and that 16 out of the 31 genes assigned to this category are differentially expressed between UE and UR cohorts *only* (i.e. not differentially expressed between TR and UR cohorts). Full results are provided in Additional file 2: Table S4

This analysis was also repeated for the exercise-specific subset of 2216 genes (i.e. exclusively associated with the exercise response relative to the training response gene list). KEGG pathways *Dilated cardiomyopathy* and *Hypertrophic cardiomyopathy* remained marginally statistically significantly over-represented (*P* = 0.0252 and *P =* 0.0472 resp.). Reactome pathways *Muscle contraction* (*P =* 1.52 × 10^−05^) and *Hemostasis* (*P =* 0.0031) and *Transmembrane transport of small molecules* (*P =* 0.0034) also remained significantly over-represented. Significant enrichment for the *Lysosome* (*P =* 0.0437) pathway, which was not found in the full exercise response gene list, was also observed. Several gene ontology sub-category terms also remained statistically significant, such as *Cytoskeletal binding* (*P =* 4.32 × 10^−05^), *Contractile fibre* (*P =* 1.12 × 10^−06^) and *Membrane lipid metabolic process* (*P =* 0.0057*).* The top twenty most significantly over-represented functional modules for this exercise-specific gene list are given in Additional file 2: Table S4 and illustrated in Fig. 2.

**Fig. 3:**
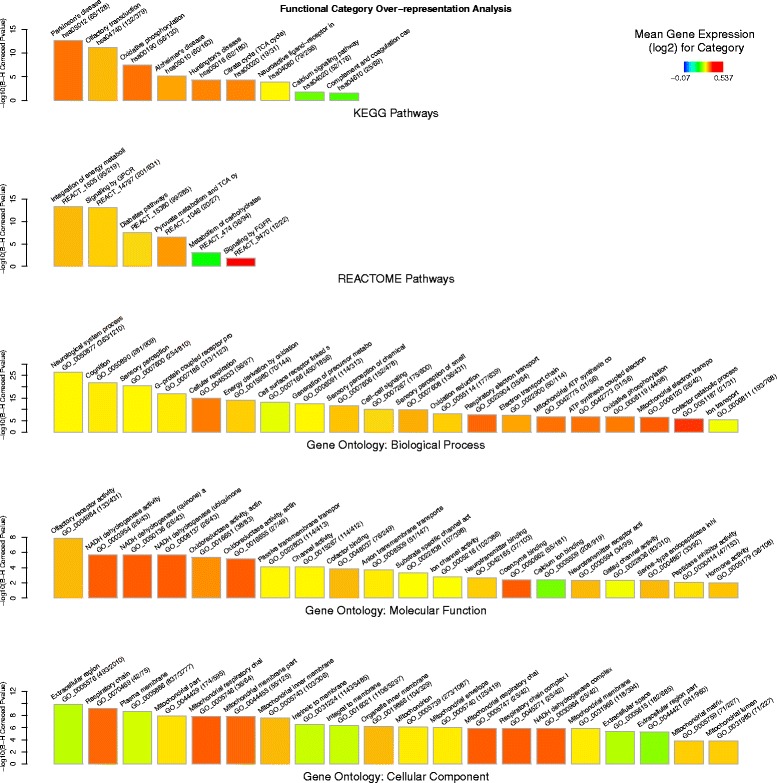
Cellular functions of the training response. Bar charts showing over-representation of functional categories (described by KEGG and REACTOME pathways and Gene Ontology: *Biological Process*, *Molecular Function* and *Cellular Component*) for the list of genes (*n* = 3405) that showed statistically significant differential expression, of at least +/− 1.25-fold, between the muscle tissue (*gluteus medius*) from the untrained rest (UR) and the trained rest (TR) Thoroughbred cohorts. Bars represent the most significant functional modules (up to 20) for each of the five annotation schemas. Bar height represents statistical significance (*−log*_*10*_ transformed Benjamini-Hochberg (B-H) Corrected *P*-value) of the over-representation, based on the EASE-score (conservative Fisher Exact t-test). Bar color represents the mean differential expression (log_2_(TR/UR) for the genes in this module (see color key). Category name, ID and size (number category genes in gene list/category size) are given above each bar. For example it can be seen that the most significant Gene Ontology:*Biological Process* is ‘Integration of energy metabolism’ and that this functional categories is up-regulated on average (orange color) and that 95 out of the 219 genes assigned to this category are differentially expressed between TR and UR cohorts. Full results are provided in Additional file 2: Table S5

For the training response gene list (3405 genes) there was significant enrichment for several KEGG and Reactome pathways related to energy metabolism (e.g. *Oxidative phosphorylation: P =* 3.59 × 10^−08^, *Citrate cycle P =* 5.12 × 10^−05^ *, Integration of energy metabolism: P =* 4.66 × 10^−14^ *, Diabetes pathways: P =* 3.10 × 10^−08^, *Pyruvate metabolism: P =* 2.93 × 10^−07^). Additionally, several KEGG and Reactome pathways related to signal transduction (e.g. *Signaling by GPCR: P =* 8.94 × 10^−14^ *, Olfactory transduction: P =* 7.68 × 10^−12^ *, Neuroactive ligand-receptor interaction: P =* 0.0001*, Calcium signaling pathway: P =* 0.0190*, Signaling by FGFR: P =* 0.0169 and *Complement and coagulation cascades: P =* 0.03) were also significantly over-represented in the training response. There was also a significant enrichment for neurodegenerative disease associated KEGG pathways (e.g. *Alzheimer’s Disease: P =* 6.57 × 10^−06^ *, Huntington’s Disease*: 4.5 × 10^−05^ *and Parkinson’s Disease:* 2.56 × 10^−13^). Gene ontology term analysis for the training response also revealed a highly significant over-representation of neurological processes (*Neurological system process: P = 4*.85 × 10^−27^ *, Cognition: P =* 1.92 × 10^−22^ *, Sensory perception: P =* 4.21 × 10^−21^) and the extracellular region (*Extracellular region: P =* 1.40 × 10^−10^). Mitochondrial related molecular functions and components were also significantly over-represented (*Mitochondrial respiratory chain: P =* 1.45 × 10^−08^ *, Mitochondrial inner membrane: P =* 2.53 × 10^−08^ *, NADH dehydrogenase*/ *Respiratory chain complex I: P =* 1.29 × 10^−06^ *, Mitochondrial proton-transporting ATP synthase complex: P =* 0.0007) in the training response gene list. The top twenty most significantly over-represented functional modules for the training response are given in Additional file 2: Table S5 and illustrated in Fig. 3.

**Fig. 4:**
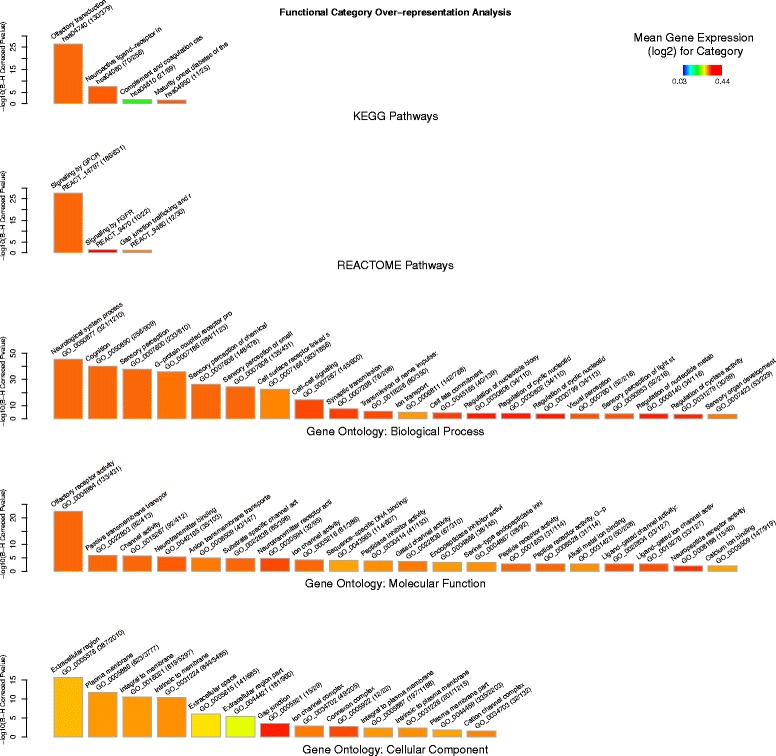
Cellular functions of the training-specific response. Bar charts showing over-representation of functional categories (described by KEGG and REACTOME pathways and Gene Ontology: *Biological Process*, *Molecular Function* and *Cellular Component*) for the list of genes (*n* = 2380) that showed statistically significant differential expression, of at least +/− 1.25-fold, between the muscle tissue (*gluteus medius*) from the untrained rest (UR) and the trained rest (TR) but not the untrained exercise (UE) Thoroughbred cohorts. Bars represent the most significant functional modules (up to 20) for each of the five annotation schemas. Bar height represents statistical significance (*−log*_*10*_ transformed Benjamini-Hochberg (B-H) Corrected *P*-value) of the over-representation, based on the EASE-score (conservative Fisher Exact T-Test). Bar color represents the mean differential expression (log_2_(UE/UR) for the genes in this module (see color key). Category name, ID and size (number category genes in gene list/category size) are given above each bar. For example it can be seen that the most significant Reactome pathway is ‘Signaling by GPCR’ (where GPCR = G protein-coupled receptors) and that it is one of the most up-regulated functional categories on average (orange color) and that 186 out of the 631 genes assigned to this category are differentially expressed between TR and UR cohorts *only* (i.e. not differentially expressed between UE and UR cohorts). Full results are provided in Additional file 2: Table S6

This analysis was repeated for the training-specific subset of 2380 genes that were uniquely associated with the training response (relative to the exercise response gene list). This training-specific gene list was also enriched for both signalling and neurological related categories (e.g. *Signalling by GPCR:P =* 2.5 × 10^−28^ *, Neurological system process: P =* 2.3 × 10^−46^). The over-representation of the cellular component: *Extracellular region* was also highly significant (*P =* 1.90 × 10^−16^). The top twenty most significantly over-represented functional modules for this training-specific subset are given in Additional file 2: Table S6 and illustrated in Fig. 4.

### Prediction of molecular interaction networks and their ‘re-wiring’ in exercise and training

**Fig. 5:**
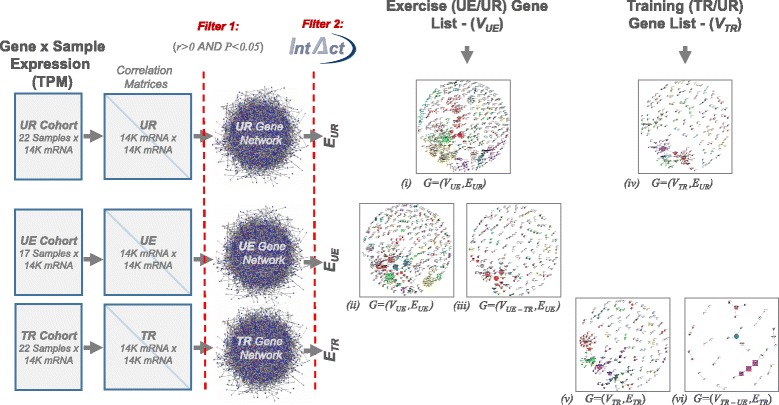
Pipeline for construction of putative dynamic PPI networks in the exercise and training responses. Each putative contextual PPI network is composed from nodes, *V*, (genes) and edges, *E*, (interactions) related to one of the experimental contexts (cohorts). For example, the putative PPI network for the exercise in the untrained rest state, *(1): G = (V*_*UE,*_ *E*_*UR*_ *)*, contains the nodes (genes) that are associated with exercise (V_UE_) and the edges (based on gene expression correlations) from the untrained rest (E_UR_) cohort that are also supported by known protein-protein interactions (PPIs) in the IntAct molecular interaction database. This can be considered the ‘ground state’ of the dynamic exercise network and can be compared to the exercise ‘active state’, *(2): G = (V*_*UE,*_ *E*_*UE*_ *)*, or the sub-network that is exclusive to exercise, *(3):G = (V*_*UE – TR*_ *,E*_*UE*_ *)* to model how these putative PPI interactions might be re-wired in the exercise response and the exercise-specific response respectively. Networks (1)–(3) are illustrated in Fig. 6(a)-(c) and networks (4)–(6) are illustrated in Fig. 7(a)-(c)

**Fig. 6:**
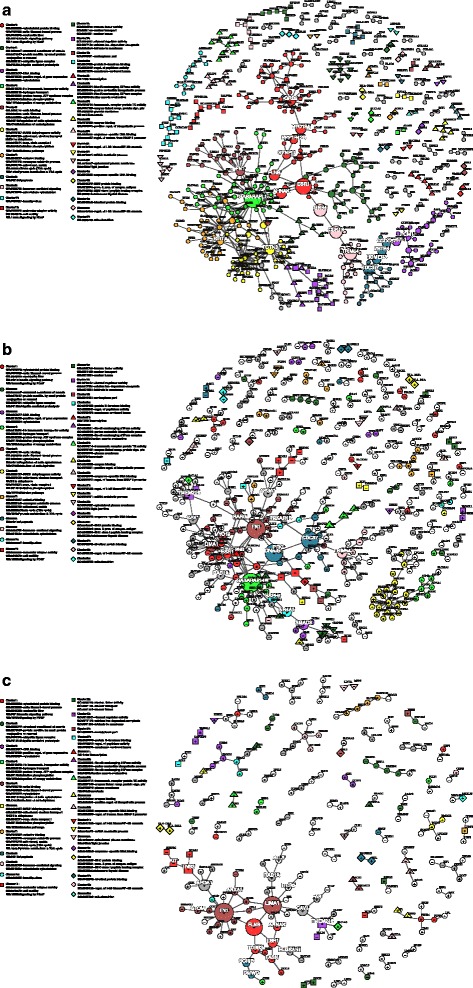
Putative dynamic PPI network for the exercise response. **a** The putative PPI network for exercise rest or ‘ground state’ contains 513 nodes (genes), after edgeless nodes were removed, and 514 edges (interactions)(see also Fig. 5 and *Methods*). This network was partitioned by Newman’s *fastgreedy* community detection (based on network topology only and with no prior information relating to gene function) into forty-three communities or node ‘clusters’. A subset of twenty-eight of these clusters had greater than two nodes (genes) and were found to be significantly enriched for at least one functional category (described by KEGG, Reactome or Gene Ontology). Node colour and shape (i.e. circle, square, up-pointing triangle and down-pointing triangle) signifies cluster membership (only functionally enriched cluster shown in legend). Node size is proportional to node ‘betweeness’ score, with the largest nodes ‘controlling’ the most network ‘traffic’ (along shortest paths). The top twenty ‘bottleneck’ nodes have white labels. **b** The network for the exercise state, which contains 426 nodes (genes), after edgeless nodes are removed, and 390 edges (interactions). Nodes (genes) are both up (‘+’ nodes) and down-regulated (‘-’ nodes) and ‘re-wired’ (loss/gain of edges) in the exercise response compared to the rest state depicted in (**a**). Cluster membership from (**a**) is transposed onto (**b**) to highlight how each cluster changes in the exercise network state (i.e. common nodes are given the same colour and shape with new nodes depicted by *uncoloured circles*). For example, it can be seen that Cluster 1 (*red circles*), which is most enriched for the ‘*Contractile fiber’* functional category becomes extensively fragmented into 10 (mostly two-node) clusters and most genes are down-regulated (19/26) signifying possible dysregulation of this functional modules in the exercise response. Conversely we also see that the Cluster 6 (*yellow circles*), which is most enriched for ‘*NADH dehydrogenase/ Mitochondrial respiratory chain complex I’*, is mostly up-regulated and remains largely intact, signifying possible coordinated up-regulation of this functional module in the exercise response. **c** Depicts the sub-network of (**b**) whose nodes (genes) are exclusive to the exercise response (i.e. not associated with the training response)

**Fig. 7:**
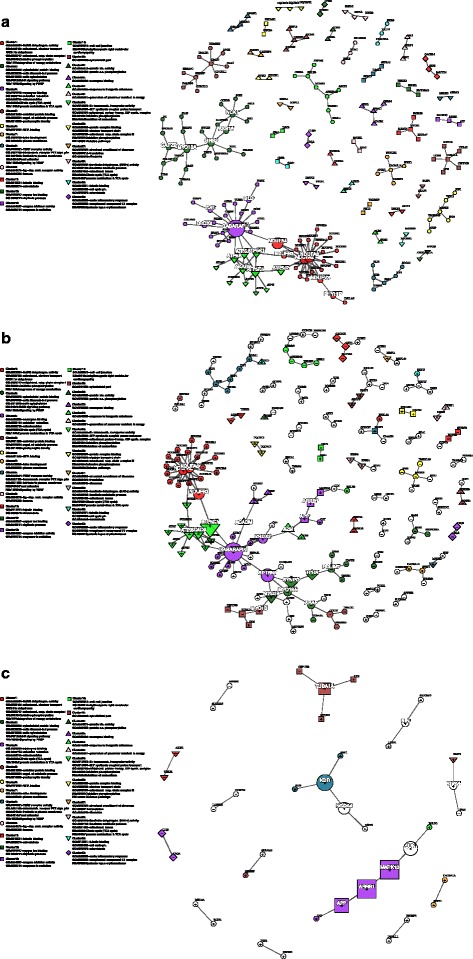
Putative dynamic PPI network for the training response. **a** The putative PPI network for untrained or ‘ground state’ contains 199 nodes (genes), after edgeless nodes were removed, and 186 edges (interactions)(see also Fig. 5 and *Methods*). This network was partitioned by Newman’s *fastgreedy* community detection (based on network topology only and with no prior information relating to gene function) into forty-three communities or node ‘clusters’. A subset of twenty-eight of these clusters had greater than two nodes (genes) and were found to be significantly enriched for at least one functional category (described by KEGG, Reactome or Gene Ontology). Node colour and shape (i.e. circle, square, up-pointing triangle and down-pointing triangle) signifies cluster membership (only functionally enriched cluster shown in legend). Node size is proportional to node ‘betweeness’ score, with the largest nodes ‘controlling’ the most network ‘traffic’ (along shortest paths). The top twenty ‘bottleneck’ nodes have white labels. **b** The network for the trained state, which contains 188 nodes (genes), after edgeless nodes are removed, and 176 edges (interactions). Nodes (genes) are both up (‘+’ nodes) and down-regulated (‘-’ nodes) and ‘re-wired’ (loss/gain of edges) in the training response compared to the untrained state depicted in (**a**). Cluster membership from (**a**) is transposed onto (**b**) to highlight how each cluster changes in the trained network state (i.e. common nodes are given the same colour and shape with new nodes depicted by uncoloured circles). For example, it can be seen that Cluster 2 (*green circles*), which is most enriched for the ‘*ErbB signaling pathway/Signaling by PDGF’* functional category becomes fragmented into 4 clusters and all visible genes (edgeless nodes are not depicted) are down-regulated. Conversely we see that the Cluster 1 (*red circles*), which is most enriched for ‘*NADH dehydrogenase/ Mitochondrial respiratory chain complex I’*, is mostly up-regulated and remains largely intact, signifying possible coordinated up-regulation of this functional module in the training response. **c** Depicts the sub-network of (**b**) whose nodes (genes) are exclusive to the training response (i.e. not associated with the exercise response)

The functional relevance of a gene or gene list in a context can only be fully appreciated by also examining how the genes, or their products, interact in that context. We therefore attempted to reconstruct context-specific dynamic PPI networks by integrating our exercise and training response gene lists and per cohort co-expression correlations with the global set of experimentally validated PPIs as described by EBI’s *IntAct* Database. First, two dynamic networks were constructed to model how the genes associated with the exercise and training responses may ‘interact’ in each of the experimental cohorts. The set of genes (network nodes) in each dynamic interaction network was confined to those genes associated with that response. The putative interactions (network edges) were inferred from the gene expression correlations in each experimental cohort. The edges in each dynamic network had two states, the *ground* state, derived from the untrained rest (UR) cohort, and the *active* state, derived by either the exercise (UE) or trained (TR) cohort. Edges were also weighted with cohort specific correlation coefficients. To better model the putative contextual protein-protein interactions (PPIs) these correlation-based interaction networks were then rigorously pruned to remove edges with no supporting experimental PPI evidence, retrieved via EBI’s *IntAct* database. An overview of this process is illustrated in Fig. 5, where output networks (i)-(vi) reflect Figs. 6 and 7, and further details are given in *Methods*. The final putative dynamic PPI network for the exercise response contained 513 nodes (edgeless nodes were removed) and 514 edges in the untrained rest (UR) state, see Fig. 6(a), and 426 nodes and 390 edges in the exercise (UE) state, Fig. 6(b).

In the exercise network the top twenty most influential genes, in terms of both *degree* and *betweeness* were identified (see Table 2 and Fig. 6). We found that the *GABARAPL1* gene *(GABA type A receptor associated protein like 1)*, which was upregulated (+1.49-fold; *P* = 6.5 × 10^−12^) in the exercise response, was the top hub and bottleneck in both the untrained rest (UR) and exercise (UE) network states. The edges of *GABARAPL1* were partially ‘re-wired’ with it gaining 14 edges, losing 22 and retaining 11 interactors in the exercise state relative to the rest state (see Additional file 1: Section 3.1). We also observed that relative influence of other hub and bottleneck genes appeared to change between network states. For example, *DNAJB* (*dnaj hsp40 member B1*), *FN1* (*fibronectin 1*) and *LIMA1* (*LIM domain and actin binding 1*) all had a greater influence in the exercise network state than the rest state as determined by both betweenness rank (*FN1* from 12th to 2nd; *LIMA1* from 73rd to 5th; *DNAJB* from 174th to 3rd) and degree rank (*FN1* from 5th to 2nd; *LIMA1* from 40th to 3rd), see Table 2 and Fig. 6. The additional edges gained by *DNAJB1* in the exercise state included a new putative interaction with, *GABARAPL1*, which effectively boosts the network-wide influence (i.e. betweeness) of *DNAJB1* by proxy.

**Table 2:** The top twenty hub (based on node degree, *D*) and bottleneck (based on node betweenness, *B*) genes in the Exercise Response network in the Untrained Rest (UR) and Untrained Exercise (UE) states

Untrained Rest				Untrained Exercise				Unique to Untrained Exercise			
*Hubs*	*D*	*Bottlenecks*	*B*	*Hubs*	*D*	*Bottlenecks*	*B*	*Hubs*	*D*	*Bottlenecks*	*B*
GABARAPL1	33	GABARAPL1	20,436	GABARAPL1	25	GABARAPL1	6905	FN1	15	FN1	888
TCTN3	21	ESR1	18,589	FN1	19	FN1	6881	LIMA1	11	LIMA1	826
NDUFA6	19	TRIM54	13,553	LIMA1	16	DNAJB1	6327	APOA1	5	FLNB	756
SIRT4	18	GNB4	13,514	NDUFA6	16	CDC37	5983	TRIM54	5	APOA1	394
FN1	16	CDK18	13,394	CDC37	14	LIMA1	2799	TMEM216	5	CAV1	346
GRB2	16	CDC37	10,875	MYO18A	13	TRIM54	2444	ESR1	5	ESR1	246
NDUFA2	15	FLNB	10,484	TMEM216	11	HSPH1	2338	BAG3	4	TMEM216	210
FASTKD3	15	AHNAK	10,125	MYO19	10	NR4A1	2210	TCTN3	4	TNS3	207
ESR1	10	MYO18A	9786	SPTBN1	9	FLNB	2123	TUBB3	4	RAB1A	158
TRIM54	9	TCTN3	9292	TRIM54	8	SMAD3	2094	FLNB	4	TUBB3	156
NCK1	9	GRB2	8676	ESR1	8	APOA1	1759	PIK3R1	4	AHNAK	120
MEOX2	8	FN1	7852	TCTN3	7	CAV1	1707	SMAD3	3	KIT	107
ATP5F1	8	HOMER3	7598	FLNB	7	RARG	1691	KIT	3	SCRIB	106
CDC37	8	ZBTB4	7364	MEOX2	6	TMEM216	1659	TNS3	3	FAS	54
TMEM216	8	CCDC57	7154	ATP5F1	6	ESR1	1603	CAV1	3	DMWD	54
ACAD9	7	CEP57L1	6650	SYNPO	6	SPTBN1	1484	CETN3	3	ITPR3	54
PCM1	7	PCM1	6593	SMAD3	5	RAB1A	1322	RAB1A	3	FASN	54
HDAC1	6	SYNPO	6545	TUBB3	5	PRKAR1B	1300	NR4A1	3	ANXA1	54
NDUFS2	6	PPP1R12B	6084	APOA1	5	PFKL	1162	HSF2	3	ELMSAN1	54
TTN	6	SIRT4	5093	PLEC	5	MYO19	1095	FASN	3	STOM	54

The top twenty hub and bottleneck genes are also provided for the sub-network that was unique to the Exercise Response (i.e. constructed from genes differentially expressed in response to exercise but not training)

We then performed a systems level analysis of the exercise response dynamic network by applying Newman’s *fastgreedy* community detection algorithm [45] to cluster the nodes in the network based only upon network topology. Twenty-eight non-trivial clusters (i.e. containing at least two genes) were detected in the untrained rest (UR) network state. The functional relevance of each of these clusters (de novo putative gene modules) was then evaluated by over-representation analysis and twenty-two clusters were found to be significantly enriched for known functional modules. The largest clusters were primarily enriched for the cellular components *Contractile fibre* (Cluster 1: 55 genes; *P* = 8.9 × 10^−07^), *Proton-transporting ATP synthase/ Mitochondrial respiratory chain complex V* (Cluster 4: 35 genes; *P =* 2.5 × 10^−10^), *NADH dehydrogenase/ Mitochondrial respiratory chain Complex I* (Cluster 6: 32 genes; *P =* 3.5 × 10^−35^) and the biological process *Pyruvate metabolism and TCA cycle* (Cluster 7: 32 genes; *P =* 2.1 × 10^−18^), see Fig. 6(a). To illustrate how these clusters are ‘re-wired’ in the exercise state we then overlaid the rest state cluster membership onto the exercise network state, by applying the same node format (colour and shape) to common nodes, see Fig. 6(b).

We observed changes in the interactions of several clusters in the exercise state relative to the rest state. The cluster enriched for *Pyruvate metabolism and TCA cycle* (Cluster 7*)* for example, which contained 32 genes that were upregulated (mean:1.41-fold) in the exercise response, became disconnected in the exercise state and had far fewer internal interactions (seven edges) relative to the rest state (39 edges), see Fig. 6(a) and (b). The top hub within this cluster, *SIRT4* (*sirtulin 4*) which was upregulated in the exercise response (+1.69-fold; *P =* 2.4 × 10^−14^), had 18 interactions in the rest state and only three interactions in the exercise state. The cluster enriched for *Contractile fibre* however (Cluster 1) became mostly downregulated (38/55 genes; mean: −1.2-fold) in the exercise state relative to rest state. It also lost many putative interactions in the exercise state (22 edges) relative to the rest state (60 edges) and became fragmented. For example, *phosphoinositide-3-kinase regulatory subunit 1* (*PI3KR1)*, which was upregulated in the exercise state (1.7-fold; 8.1 × 10^−12^), formed a new independent sub-cluster. This new cluster included new putative interactions with Cluster 31 members *STAT3* (*signal transducer and activator of transcription 3*) and *SP1* (*Sp1 transcription factor*) which were also upregulated in the exercise response 1.35-fold (*P* = 2.8 × 10^−12^) and 1.26-fold (*P =* 0.0056) respectively (see Fig. 6b). Other clusters, however, were upregulated in a more coordinated manner in the exercise state (i.e. retained many intra-cluster edges). For example, the cluster enriched for *NADH dehydrogenase/Mitochondrial respiratory chain complex I* (Cluster 6), which is upregulated in the exercise response (mean:1.35-fold), retained 23 of its 52 rest state edges in the exercise state. Most these retained edges (20/23) were between genes that encode subunits of *NADH dehydrogenase/ Respiratory chain complex I*. The top hub in this cluster, in both the rest (19 edges) and exercise state (16 edges), was *NDUFA6* (*NADH: ubiquinone oxidoreductase subunit A6*). This cluster (Cluster 6) also became detached from the main network in the exercise state (along with 5 *ATP synthase complex/ Respiratory chain complex V genes* from Custer 5), see Fig. 6(b), and its expression appears to be more independent from the rest of the network relative to rest state (where it previously had seven external edges).

Some clusters became more central in the exercise state relative to the rest state, as determined by betweeness. For example, two genes in the cluster enriched for *Apoptosis* (Cluster 8), namely *CDC37* (*cell division cycle 37;* −1.27-fold; *P =* 1.92 × 10^−07^) and *DNAJB* (*DnaJ Hsp4 member B1;+*4.27-fold; *P =* 3.59 × 10^−14^), gained a greater network influence in the exercise state relative to the rest state, being promoted from 174th and 6th to 3rd and 4th respectively in rank based on betweenness (see Table 2). Additionally, two genes in the cluster enriched for *Cytoskeleton* (Cluster 5)*, FN1* (*fibronectin 1; +*1.29-fold; *P =* 0.0018) and *LIMA1* (*LIM domain and actin binding 1;+*1.55-fold; *P =* 9.7 × 10^−12^), were also promoted from 8th and 73rd to 2nd and 5th respectively (see Table 2).

In the exercise-specific network (genes associated with the exercise but not the training response) the cluster enriched for *Cytoskeleton* (Cluster 5) was the largest upregulated cluster (see Fig. 6(c)). This upregulation also appears to be coordinated as it retained a majority of its intra-cluster interactions (14/26 edges). Genes in this cluster, *FN1* and *LIMA1,* also became the top two bottlenecks in this exercise-specific network. *FLNB* (*filamin B*), which was also upregulated in exercise-specific response (+1.66-fold; *P* = 1.1 × 10^−09^), shared edges with both *FN1* and *LIMA1* and was the third most influential bottleneck in the exercise-specific response network.

In the training response putative dynamic PPI network, which was constructed as for the exercise response, there were 199 nodes and 186 edges in the untrained state (UR) and 188 nodes and 176 edges in the trained (TR) state (see Fig. 7). The top twenty most influential genes, determined by betweenness and degree, are given in Table 3 and highlighted in Fig. 7. As in the exercise response network, *GABARAPL1* was the top bottleneck in the training response network both in the untrained state and trained state and was significantly upregulated (+1.37-fold; *P =* 0.0001) in response to training. *GABARAPL1* was partially ‘re-wired’ in the trained state (15 edges), gaining three and losing seven interactions relative to the untrained state (19 edges) of the network (see Additional file 1: Section 3.2). The highest degree node in both states was *NDUFA6* (*NADH ubiquinone oxidoreductase subunit A6*), which was upregulated (+1.36-fold; *P =* 0.0007) in the training response and encodes a subunit of *NADH dehydrogenase/Mitochondrial respiratory complex I*. Other top bottlenecks across both the untrained and trained network states included several genes that also encode subunits of this complex (*NDUFA2, NDUFA4, NDUFA6, NDUFB3, NDUFV3*) and *ATP synthase complex*/*Respiratory chain complex V* (*ATP5A1, ATP5B, ATP5C1, ATP5F1, ATP5J2*). These genes were all significantly upregulated in the trained cohort (mean:1.38 fold; *P =* 0.0018).

**Table 3:** The top twenty hub (based on node degree, *D*) and bottleneck (based on node betweenness, *B*) genes in the Exercise Response network in the Untrained Rest (UR) and Trained Exercise (TR) states

Untrained Rest				Trained Rest				Unique to Trained Rest			
*Hubs*	*D*	*Bottlenecks*	*B*	*Hubs*	*D*	*Bottlenecks*	*B*	*Hubs*	*D*	*Bottlenecks*	*B*
NDUFA6	20	GABARAPL1	819	NDUFA6	19	GABARAPL1	2137	KDR	3	MAPK10	6
GABARAPL1	19	NDUFA4	459	GABARAPL1	15	ATP5C1	1631	TUBA1A	3	ARRB1	6
NDUFA2	15	NDUFA6	432	NDUFA2	14	SPTBN1	1135	NCOA4	2	KDR	5
ATP5F1	8	NDUFA2	320	ATP5F1	8	NDUFB3	1067	NIN	2	JUN	4
GRB2	7	GRB2	177	SPTBN1	5	PRDX2	935	JUN	2	APP	4
CDC37	6	MYO18A	158	ACAD9	5	NDUFA2	933	MAPK10	2	NCOA4	3
ACAD9	6	ATP5F1	125	MYO19	5	SYNPO	842	ARRB1	2	TUBA1A	3
NCK1	6	NDUFV3	98	MYO18A	5	A2M	814	APP	2	NIN	1
TUBA1A	5	ATP5A1	88	MAPK6	4	NDUFA6	777	RIPK4	2	RIPK4	1
MYO18A	5	ASB16	85	ARAF	4	MYO18A	608	TNKS2	1	TNKS2	0
CACNA1A	4	ATP5B	82	LUZP1	4	ARAF	565	IPO4	1	IPO4	0
ATP5B	4	NCK1	82	KDR	4	MYO19	549	EGLN3	1	EGLN3	0
HSF2BP	4	CAPZB	78	PRDX2	4	APP	509	LUZP1	1	LUZP1	0
ATP5C1	4	ACAD9	76	PDLIM7	4	ACAD9	401	C1QB	1	C1QB	0
MAPK6	3	ATP5J2	76	SYNPO	4	ATP5F1	395	C1QA	1	C1QA	0
EGLN3	3	ATP5C1	72	ATP5C1	4	TPRN	323	SPRY1	1	SPRY1	0
HDAC1	3	NDUFB3	64	HDAC1	3	PDLIM7	320	FGF2	1	FGF2	0
CAPZB	3	PHYHIP	50	SPRY1	3	ACADM	320	FGFBP1	1	FGFBP1	0
KDR	3	DLD	50	NDUFS2	3	ARRB1	246	CACNA1A	1	CACNA1A	0
NDUFA4	3	PRDX2	50	CDC37	3	MYO1B	246	DYNLL1	1	DYNLL1	0

The top twenty hub and bottleneck genes are also provided for the sub-network that was unique to the Training Response (i.e. constructed from genes differentially expressed in response to training but not exercise)

We observed that the network influence, as determined by hub or bottleneck status, of several genes changed in the trained state (TR) relative to the untrained network state (UR). *SPTBN1 (spectrin beta, non-erythrocytic 1),* which was upregulated in response to training (+1.38-fold; *P =* 0.0052), was promoted in rank from 55th in the untrained state, to 3rd top bottleneck in the trained network state. *SPTBN1* maintained its edge with *GABARAPL1* but also appears to have undergone a ‘re-wiring’ of other edges. For example, *SPTBN1* gained edges with ‘unconventional’ myosins (as opposed to Class II myosins that are directly involved in muscle contraction [70]), *MYO19* and *MYO18A,* and an actin-associated protein gene *SYNPO* (*synaptopodin*). Conversely, the *GRB2* (*growth factor receptor bound protein 2*) gene, which was downregulated (−1.33-fold, *P =* 0.0002) in the training response, became less influential in the trained (TR) state relative to the untrained network state, dropping from 5th to 49th place in the betweenness-based rankings.

Community analysis, performed as above, uncovered twenty-three clusters within the training response network untrained state. Of these, twelve non-trivial (more than two genes) clusters were significantly enriched for one or more known functional category (see Fig. 7(a)). Three of the clusters uncovered were interconnected (six edges) and were enriched for *NADH dehydrogenase/Mitochondrial respiratory complex I* (Cluster 1), *ATP synthase complex*/ *Mitochondrial respiratory complex V* (Cluster 34) and *Pyruvate metabolism and TCA cycle* (Cluster 3)*.* The genes in these clusters were mostly upregulated (i.e. 32/36, 12/14 and 10/20 genes respectively) in the training response (see Fig. 7(b)). These clusters also appeared to be upregulated in a coordinated manner due to the retention of many intra-cluster (internal) edges in the trained network state relative to the untrained state. The cluster enriched for *NADH dehydrogenase/ Mitochondrial respiratory complex I* (Cluster 1) retained 32/36 edges, the cluster enriched for *ATP synthase complex*/*Respiratory complex V* (Cluster 34) retained 12/14 edges and the cluster enriched for *Pyruvate metabolism and TCA cycle* (Cluster 3) retained 10/20 edges (see Fig. 7(b)). Furthermore, these three clusters also retained 5/6 inter-cluster edges (between clusters) in the trained network state, implying coordination at the system level also. Another six-gene cluster, enriched for *Signaling by VEGF/Focal adhesion* (Cluster 6), was entirely upregulated (mean: 1.59-fold) in a coordinated manner (retained all intra-cluster edges) in the training state but remained independent from the other clusters (see Fig. 7(b)). In contrast to the above clusters, the cluster enriched for *ERBB Signaling pathway/Signaling by PDGF* (Cluster 2), which was the largest cluster (24 genes), became entirely downregulated (mean: −1.6-fold) and dis-coordinated (retained only 6/25 intra-cluster edges) in the trained network state compared to the untrained network state (see Fig. 7(b)). Another 10-gene cluster, which was enriched for *Unfolded protein binding/ Postsynaptic density* (Cluster 5)*,* contained heatshock protein genes *DNAJB1(DnaJ hsp40 member B1), HSF2BP (heat shock transcription factor 2 binding protein)* and co-chaperone genes *CDC37* (*cell division cycle 37*) and *CDC37L1* (*cell division cycle like 1*). This cluster became partially downregulated (five genes; −1.54-fold) and upregulated (five genes; mean: +1.38-fold) and ‘re-wired’ (lost seven edges and gained one) in the training response.

The training-specific network (genes associated with the training response but not the exercise response) contained 25 putative interactions and little modular structure (only two modules with a diameter > 2) (see Fig. 7(c)). The largest contained six genes (including three genes from Cluster 13), which were all upregulated (mean: +1.3-fold) in the training response, namely *APP* (*amyloid beta precursor protein*)*, ARRB1*(*arrestin beta 1*), MAPK10 (*mitogen-activated protein kinase 10*), *JUN* (*jun proto-oncogene AP-1 transcription factor subunit*), *EGLN3* (*egl-9 family hypoxia inducible factor 3*), and *CAT* (*catalase*) (Fig. 7(c))*.* Another module containing five genes was also upregulated in response to training (mean:1.49-fold) and contained part of the cluster enriched for *Cytoskeleton* (Cluster 8), namely *KDR* (*kinase insert domain receptor, a.k.a. VEGFR-2*)*, NRP1* (*neuropilin 1*) and *FLT4 (fms related tyrosine kinase 4, a.k.a. VEGFR-3)*. This cluster also gained two additional interactions in the training response, *NCOA4* (*nuclear receptor coactivator 4*) and *USP43* (*ubiquitin specific peptidase 43*), that were present in this training-specific sub-network.

## Discussion

Using a transcriptome-wide RNA-seq approach in a large cohort of active racehorses, we have for the first time generated a comprehensive set of genes that are differentially expressed in Thoroughbred skeletal muscle in response to both exercise and training. We have also developed a novel computational strategy to integrate these data with publicly available, experimentally validated PPIs in a bid to model the contextual interactome, its functional modules and how it may respond to exercise and training. We observed that approximately one third of genes that were diffientially expressed in response to a single bout of exercise accumulate with training leading to new baseline levels of gene expression, suggesting that increases in aerobic capacity may be brought about mainly through this mechanism. Therefore, consecutive bouts of exercise training seem to result in a priming of the skeletal muscle transcriptome for the demands of the next exercise bout. We have also demonstrated that this may further lead to an extensive ‘re-wiring’ of the molecular interactome in both exercise and training and have identified key genes and functional modules that may be involved in controlling and mediating these responses.

In this study the training period was six months, therefore it is possible that some of the changes in gene expression attributed to training may be age-related. As this study was undertaken in collaboration with an active racing yard, which offers considerable advantages in terms of cohort size, consistent exercise/training regimen and consistent environmental factors, such as nutrition, certain controls, such as untrained mature horses were not possible to obtain at the same time point as the TR samples. However, training standards ensured that all time points in the study (>513 days) were well beyond the end of the pubescent period for Thoroughbred horses (<450 days) [71]. Furthermore, a small subset of the UR cohort (*N* = 4) was sampled after a two-week period of detraining, Detrained Rest (DR), and while there was some overlap among DE genes (28 genes), there was very little difference in terms of overall DE (144 genes after correction for multiple comparisons) between these two cohorts (See Additional file 1: Section 5 and Additional file 2: Table S7.)

Our initial functional over-representation analyses supported a central role for modules related to energy metabolism in both the exercise response and training response. Interestingly, several functional modules of this type, which were common to both, were more significantly associated with the training response than the exercise response in terms of module significance (see Fig. 1 vs. Fig. 3). For example, the exercise response was enriched for *Integration of energy metabolism* with a significance level of *P* = 4.97 × 10^−11^; however, this increased to a significance level of *P =* 4.66 × 10^−14^ in the training response. We also observed a similar trend for *Pyruvate metabolism and TCA cycle* (from *P =* 0.0204 to *P =* 2.93 × 10^−07^) and *NADH dehydrogenase/Mitochondrial respiratory chain complex I* (from *P =* 0.0017 to *P =* 1.29 × 10^−06^). Furthermore, some functional modules related to energy metabolism were only found to be significantly over-represented in the training response gene list. These included the *Citrate cycle* (*P =* 5.12 × 10^−05^) and the respiratory chain component *Proton-transporting ATP synthase / Respiratory chain complex V* (*P =* 0.0007). These results support the hypothesis that trained skeletal muscle may exhibit an unstimulated transcriptome inherently primed to respond to the energy demands of exercise. More generally this information also provides support for the view that ‘accumulative’ changes play a role in the adaptive response.

There is also some evidence that epigenetic regulation may play a role in modulating these transcriptomic adaptations. For example, four of the five genes reported by Barrès and colleagues to be hypomethylated and expressed in human skeletal muscle in response to acute exercise, namely *PPARD (peroxisome proliferator activated receptor delta)*, *PPARGC1A (PPARG coactivator 1 alpha)*, *PDK4 (pyruvate dehydrogenase kinase 4)* and *CS (citrate synthase)*, [72] were significantly upregulated either in the exercise response (*PPARD*:+1.97-fold; *P =* 1.2 × 10^−13^, *PPARGC1A:*+10.17-fold*; P =* 7.25 × 10^−26^, *PDK4*:+2.17-fold; *P =* 0.0092) or the training response (*CS*:+1.33-fold; *P =* 1.40 × 10^−05^). In particular, we found that *PPARGC1A*, which was reported by Barrès and colleagues to be hypomethylated in response to acute exercise*,* was the 6th most significantly upregulated gene (10-fold, *P =* 7.25 × 10^−26^) in the exercise response. PPARGC1A was also shown previously to be up-regulated at the protein level after high intensity exercise [73]. PPARGC1A directly links external physiological stimuli and mitochondrial biogenesis [74], regulates muscle fibre type [75] and is associated with endurance exercise [76]. It has also been shown to mediate the epigenetic regulation of insulin secretion [77] and regulate oxidative energy metabolism during exercise in equine skeletal muscle [30]. Our previous work has also supported the involvement of PPARGC1A in the adaptation of equine skeletal muscle to training [32, 78].

Another gene highlighted by Barrès et al., *CS,* which encodes citrate synthase that catalyses the synthesis of citrate from oxaloacetate and acetyl coenzyme A, was upregulated in the TR cohort. This may contribute to the trained cohort’s constitutive priming for exercise. Indeed, increased CS activity has previously been reported in trained human [79] and equine [80, 81] muscle and is a validated biomarker for skeletal muscle mitochondrial density and oxidative adaptation to a training [82].

We also observed that several functional modules uncovered by over-representational analysis were exclusively associated with either the exercise response or the training response. Only the exercise response, for example, was significantly enriched for functional modules related to muscle contraction. The largest module of this type was *Contractile fibre* (*P =* 6.35 × 10^−11^) and contained a majority (44/56) of downregulated genes. This observation is in line with the previous finding of an inhibitory effect of NAD+/NADH ratio on MYOD1 regulated gene expression and differentiation [83]. NAD+/NADH ratio is generally found to increase in muscle cells post-exercise in animals but, curiously, not in humans [84, 85]. We also observed that MYOD1, which is known as a ‘master switch’ of muscle cell differentiation [86], was significantly downregulated at the transcript level (−1.9-fold; *P =* 0.0008) but only in the training response, which suggests that it may form part of the adaptive response to training.

We also found that the *Hemostasis* pathway was exclusively significantly over-represented (*P* = 3.76 × 10^−05^) in the exercise response, it being mostly (46/69 genes) upregulated in exercise relative to rest cohort. This response included several upregulated growth factors, such as *EGF, Epidermal Growth Factor,* (+1.73–fold; *P =* 6.80 × 10^−08^), *TGF-beta 2*, *transforming growth factor beta 2* (+1.29-fold; *P =* 0.0006) and *VEGFA, vascular endothelial growth factor A,* (+2.33-fold, *P =* 4.67 × 10^−19^), *VEGFC, vascular endothelial growth factor C* (+1.79-fold, *P =* 6.65 × 10^−10^) and *VEGFD vascular endothelial growth factor D,* (+1.48-fold, *P =* 5.77 × 10^−09^). This observed association of hemostasis with the exercise but not the training response gene list aligns with recent findings in human of a transient hypercoagulability state post-exercise, particularly in untrained individuals, that is largely reversed in trained individuals [87].

Conversely, the training response gene list was also exclusively enriched for certain functional modules, such as the pathways *Signalling by GPCR* (*P =* 1.22 × 10^−17^) and *Neurological systems processes* (*P = 4*.85 × 10^−27^), that were not associated with the exercise response. G-protein-coupled receptors (GPCRs) mediate physiological responses to hormones, neurotransmitters and environmental stimulants [88]. The GPCR family also includes opioid receptors (ORs), discussed in further detail below in relation to the network analysis results.

Interestingly, we also observed over-representation of several disease related pathways in both our exercise and training associated gene lists. For example, both gene lists were enriched for Reactome’s *Diabetes pathways*, which were more associated with the training response (*P =* 3.10 × 10^−08^ *)* than the exercise response (*P =* 3.57 × 10^−06^). It is recognized that regular physical exercise, particularly aerobic and resistance exercise, has considerable health benefits for people with both type 1 and type 2 diabetes [89]. Our over-representation results also appear to provide some support for a relationship between our exercise phenotypes and diabetes and it would be interesting, in future studies, to investigate the extent of this mechanistic link. We also observed that several neurodegenerative disorders, namely Alzheimer’s Disease (AD), Huntington’s Disease (HD) and Parkinson’s Disease (PD), were amongst the most significantly enriched KEGG categories associated with both the exercise response and the training response. The links between these neurodegenerative diseases and mitochondrial dysfunction have been well established [90] and the over-representation of these pathways may be due in part to the effect that intense exercise has on mitochondrial function in skeletal muscle. Interestingly, an early deficit of synaptic mitochondria and motor function has been associated with AD [90, 91], physical exercise has been shown to be protective in both AD and PD [92] and increased mitochondrial biogenesis has recently been shown to improve symptoms in HD [93]. Our results also appear to reflect this mitochondrial-driven mechanistic relationship between exercise and this group of neurodegenerative disorders.

Standard over-representation analysis, as performed here, is based on well-established, carefully curated, functional annotations of genes and gene products (e.g. KEGG, Reactome, gene ontology). However, these resources are far from complete. For example 85% of Ensembl genes are not mapped to a KEGG pathway [34], and some genes may have additional undescribed roles [94]. For example, the kappa opioid receptor (KOR) signalling pathway, which we discuss below, was not described in sufficient detail by KEGG (only as part of the *Neuro-active ligand receptor pathways*) or fully labelled by Reactome (covers the *mu* but not the *kappa*-OR, see [95]) to be identified by our above over-representation analysis. Furthermore, even well established and fully annotated pathways/complexes may behave in an unexpected manner in a particular context (e.g. cell type, species or experimental condition) [96]. In addition, due to the group-wise nature of over-representation analysis the chief drivers or regulators of biological processes may not always be apparent (nor are these, of course, necessarily highlighted by basic statistical association of expression gene). These limitations, however, may be somewhat overcome by employing a less supervised, network analysis-based approach.

When we constructed our putative contextual PPI networks for exercise and training we first identified the most influential genes in the various states as determined by hub and bottleneck status (see Tables 2 and 3). Interestingly, we found that some nodes appeared to gain influence in response to exercise and/or training relative to the untrained rest network state. In the exercise response network for example, *FN1* (*fibronectin*) became more influential in the network after exercise relative to the untrained rest state (promoted from 11th to 2nd top bottleneck). *FN1* is involved in cell adhesion, migration, growth and differentiation [97], and interestingly, has previously been used as an indicator of myofibre damage, such as may occur after intense exercise [98]. Another gene that appeared to become more influential in the post-exercise network state, *LIMA1* (*LIM Domain and actin binding 1*), has been shown to stabilize the vascular capillary network in vitro [99]. This information, and the fact that neither *FN1* nor *LIMA1* expression was associated with the training response, might indicate involvement in muscle or microvascular recovery and repair following intense exercise. Continuing in this theme, the top differential bottleneck gene *DNAJB1* (*DnaJ Hsp40 Member B1*), which was significantly (*P =* 3.6 × 10^−14^) upregulated (>4-fold) in response to exercise, has previously been found to be upregulated in skeletal muscle in response to heat shock [100]. Interestingly this is one of the few genes whose expression is also significantly reversed in the training response (−1.6-fold, *P =* 0.0005). This also supports previous studies which reported the significant upregulation (between 1.5 fold and 4.8-fold) of heat shock proteins both immediately after and 4 h post-exercise in Thoroughbred horses [27]. Together these data suggest that these genes may be involved in the stress response induced by intense exercise in the untrained cohort.

Several other genes, such as *SIRT4 (sirtulin 4)*, *GRB2 (growth factor receptor bound protein 2)*, *GNB4* (*G protein subunit beta 4*) and *CDK18* (*cyclin dependent kinase 18*), became less influential in the exercise network state relative to untrained rest network state (i.e. their hub/bottleneck ranks were all down-graded after exercise). Although *SIRT4* had far fewer edges in the exercise state relative to rest state (4 vs. 18 edges) it was highly significantly upregulated (+1.69-fold; *P =* 2.4 × 10^−14^) in the exercise response. This perhaps suggests a shift in function in response to exercise. SIRT4 has been shown to associate with mitochondria and to have ‘strong and reproducible’ ADP-ribosyltransferase activity [101]. *SIRT4* is also required for protection against cell death caused by nutrient starvation via a role in the maintenance of mitochondrial NAD+ levels [102]. Interestingly, the only novel edge that was gained by *SIRT4* in the exercise response was with *SLC25A5 (solute carrier family 25 Member 5),* which was also highly significantly upregulated in the exercise response only (+3.2-fold; *P =* 5.3 × 10^−21^ *)* relative to untrained rest. SLC25A catalyses the exchange of mitochondrial ATP with cytoplasmic ADP across the mitochondrial inner membrane [103]. *SIRT4* also retains an edge with *ETHE1* (*persulfide dioxygenase*), which was also upregulated in the exercise response (+1.3-fold; *P* = 9.5 × 10^−08^). This mitochondrial matrix sulphur dioxygenase is involved in catabolizing hydrogen sulphide [104], a by-product of energy metabolism that inhibits mitochondrial functioning [105]. Together these data suggest that the re-wiring of *SIRT4* interactions in the exercise state may be part of the exercise-induced stress response and may contribute to the maintenance of metabolic homeostasis in mitochondria.

In the trained network, we also observed evidence of a re-wiring of key hubs. *SPTBN1* (*spectrin beta chain non-erythrocytic 1*), which was upregulated in the training response (+1.38-fold; *P =* 0.0052), appears to gain network influence in the trained network state (promoted in rank from 55th to 3rd top bottleneck). Functionally, SPTBN1 is involved in secretion, interacts with calmodulin in a calcium-dependent manner and has been isolated from post-synaptic density preparations [106]. Furthermore, *SPTBN1* has previously been shown to be associated with skeletal muscle adaptation, specifically during the torpor phase of hibernation in mammals [107]. Metabolic adaptation and the prevention of muscle atrophy during hibernation induced starvation have also been previously linked to athletic endurance adaptions [108]. In further support of a mechanistic connection between these seemingly disparate physiological states, the reduced expression of the *myostatin* (*MSTN*) gene, referred to as the Thoroughbred ‘speed gene’ [13], has also been associated with hibernation in ground squirrels [109]. Similarly, another gene, *PDK4* (*pyruvate dehydrogenase kinase*), has also been previously associated with hibernation in mammals as well as intense exercise in Thoroughbred horses [31, 110]. These data suggest perhaps that a similar biological mechanism may be involved both in the process of muscle growth during training and prevention of muscle wasting during prolonged inactivity and nutrient starvation.

The inferred contextual network may also provide some additional mechanistic insight into the activity of *SPTBN1* in the context of skeletal muscle adaptations to training. In the trained network, the *SPTBN1* bottleneck mediates the ‘flow of information’ between the module containing *GABARAPL1* and the module containing unconventional myosins, *MYO19* and *MYO18A,* and *SYNPO* (*synaptopodin*). *MYO19*, which was upregulated in both the exercise (+1.5-fold; *P =* 3.9 × 10^−08^) and trained (+1.3-fold; *P =* 0.0046) responses, is known to be involved in mitochondrial motility [111]. Furthermore, *SYNPO*, which was downregulated in both the exercise (−1.66-fold; *P =* 6.7 × 10^−14^) and trained (−1.59-fold; *P = 7*.2 × 10^−05^) responses, is known to be part of the post-synaptic density [112]. ATP and Ca^2+^ flux is particularly important at the synapse, and mitochondria are usually present in pre-synaptic terminals [113]. Taken together the above ‘re-wiring’ of *SPTNB1* interactors may possibly reflect changes to the neuromuscular junction that occurs in response to exercise and training and may also suggests an increased influence of *SPTNB1* in the training response.

Although we observed *MSTN* to be highly significantly downregulated in both the exercise (−5.9-fold; *P =* 1.4 × 10–^19^) and training (−6.4-fold; *P =* 1.5 × 10^−06^) responses, it was absent from our putative contextual PPI networks as none of its contextual (correlation-based) edges were supported by experimentally validated PPIs. This may be partially explained by the stringency of our protocol (e.g. the exclusion of inverse co-expression correlations, inclusion only of PPIs supported by IMEX standard) and also lack of recorded interaction evidences for MSTN, due in part to that fact the MSTN ligand generally interacts with membrane-bound proteins, which are under-represented in PPI databases [114]. However, by examining the larger non-validated correlation-based networks we observed some evidence of a change in *MSTN*-related activity in the exercise and training responses. We saw a reduction in *MSTN* edges in both the exercise and training network states relative to the untrained rest state. Also, approximately 20% (43/231) of exercise-induced *MSTN* edges were also observed in the trained state, suggesting that there may be some sustained adaptation of the MSTN-related signalling network in the trained cohort, although, of course, further support is required to substantiate this. Interestingly however, this list of 43 genes includes *PVALB* (*parvalbumin*), which was the most downregulated gene in both the exercise response (−25.43-fold; *P =* 3.5 × 10^−20^) and the training response (−33.27-fold; *P =* 7.8 × 10^−10^). *PVALB* is involved in calcium sequestration after muscle contraction to support muscle relaxation and a recognized fast-twitch phenotype gene [115].It has also been found that double-knockout PV−/− mice have prolonged muscle relaxation time, due to the maintenance of muscle Ca^2+^ levels, but also generate ~40% more force per contraction than PV +/− and WT animals [116].

By far the most influential gene, as determined by hub and bottleneck analysis, across both the exercise and training response networks was *GABARAPL1* (*GABA type A receptor associated protein like 1*), where it ranked as top bottleneck across all network states (Tables 2 and 3). We found *GABARAPL1* to be highly significantly upregulated (+1.49-fold; *P =* 6.5 × 10^−12^) in response to exercise and significantly upregulated (+1.37-fold; *P =* 0.0001) in the trained cohort compared to the untrained cohort.

The product of *GABARAPL1* is a ubiquitin-like modifier associated with the autophagosome and known to cause an increase in cell-surface expression of the G protein-coupled receptor (GPCR) *kappa* type opioid receptor (KOR), through facilitating anterograde trafficking of the receptor [117]. KOR is the product of the *OPRK1* gene. In our data *OPRK1* mRNA expression showed no association with the exercise response, however it was significantly upregulated (+1.74-fold; *P =* 0.0075) in the training response. Taken together this information supports the model of an initial post-translational upregulation of the KOR cell-surface expression by GABARAPL1 in response to exercise and then, upon adaption, a transcriptional upregulation in the trained cohort. However, the downstream effect of the putative upregulation of the KOR in the context of exercise and training still needs to be clarified. KOR mediated signal transduction is known to stimulate several kinase cascades depending upon the specific activating ligand [118]. For example, dynorphin activation of KOR has been shown to be involved in the stress response (e.g. to stressors such as forced swim) in mice [119]. This same ligand-receptor interaction has also been associated with pro-addictive behaviour [120]. Further work is needed in this context, but we hypothesize that the regulation of aversive/motivational behaviour by KORs may play a role in the adaptation of the organism to regular and sustained bouts of intense exercise.

We can also attempt to extract additional mechanistic and functional insight into *GABARAPL1* in the context of exercise and training from community analysis. *GABARPL1* is a member of the exercise network cluster that is significantly enriched (*P =* 2.56 × 10^−10^) for the *Proton-transporting ATP synthase/Mitochondrial respiratory chain complex V (MRC complex V)*. This cluster also has four putative interactions with the cluster that is significantly enriched (*P =* 9.69 × 10^−38^) for *NADH dehydrogenase*/ *Mitochondrial respiratory chain complex I* (*MRC complex I*) (see Fig. 6(a)). In the exercise response, we observed a general upregulation of the genes in both clusters and a general disturbance in the rest of the network (see Fig. 6(b)). These clusters became ‘re-wired’ but remained largely intact except for a major de-coupling of the *MRC complex I-enriched* cluster (along with genes encoding members of *MRC complex V*) from the rest of the network in the exercise state (see Fig. 6(b)). We observed that correspondingly enriched clusters (i.e. also enriched for MRC complexes I and V) were also present and upregulated in the trained state network (see Fig. 7(b)). However, in this case, the upregulation of these clusters was better coordinated with the rest of the network and was not accompanied by the similar de-coupling. Interestingly, this apparent ‘de-coupling’ of *MRC complexes I* and *V* during exercise, may be a reflection of the recognized physiological response to oxidative stress known as *uncoupling* and mediated by UCP3 in skeletal muscle [23]. This is also supported by the highly significant upregulation of *UCP3* (+4.8-fold; *P =* 7.6 × 10^−22^) in the exercise response. Interestingly, we did not see the differential expression of *UCP3* in the training response (although we did observe a significant increase (+1.27-fold; *P =* 0.0007) of *UCP1* and a decrease of *UCP2* (+1.47-fold; *P =* 0.0175)).

It has been suggested that *NADH dehydrogenase*/ *Mitochondrial respiratory chain complex I* activity is among the major rate-controlling steps in mitochondrial oxidative phosphorylation [121], thus its coordinated upregulation in the training response may represent a significant adaption for the overall process of oxidative phosphorylation. The central position of *GABARAPL1* in both the exercise and trained networks may suggest a driver/regulatory role in these responses, perhaps mediated in part via its control of kappa OR cell-surface expression, and downstream effects may include the altered regulation of the components of the mitochondrial respiratory chain. Previous studies have shown that the knock-down of *GABRAPL1* leads to many cellular bioenergetic changes, including increased basal oxygen consumption rate, increased intracellular ATP (possibly due to OR-mediated dysregulation of adenylyl cyclinase/cAMP signalling), increased total glutathione, and an accumulation of damaged mitochondria [117]. It has also recently been reported that the dysregulation of autophagy, in which GABARAPL1 has a central role, is linked to several neuromuscular disorders in human [122]. In the context of exercise and training, autophagy has already been shown to be activated by high-intensity, but not low-intensity, exercise in human [123] and to be involved in the maintenance of muscle mass in *Atg7 (autophagy related 7)* knock-out mice [124]. It has also been proposed that autophagosomal proteins, of which GABARPL1 is one, may act as sensors for oxidative stress (via redox signalling) and mediate cross-talk between this and other cellular processes such as mitochondrial turnover [125]. This latter relationship, in particular, would explain the tight coupling of *GABARAPL1*, *NADH dehydrogenase/ Mitochondrial respiratory chain complex I* and *Proton-transporting ATP synthase/Mitochondrial respiratory chain complex V* clusters that we observed in the exercise (rest state) and training networks. There is already some experimental evidence to support a role for autophagy in muscle cell homeostasis during exercise [126, 127]. Furthermore, there is support for GABARAPL1 being directly involved in mediating the autophagic energy stress response in heart muscle, involving both mitophagy and glycophagy, via its interaction starch binding domain 1 (STBD1) protein [128]. Our results indicate that *GABARAPL1* may play a central role in the adaption to oxidative stress induced by intense exercise and may also play a key role in the adaptive response to training, perhaps in part via a role in the recycling and upregulation of mitochondria via mitophagy.

The training response network also contains evidence of ancillary adaptations. For example, a small three-gene module, containing *APP (amyloid beta precursor protein), ARRB1(arrestin beta 1)* and *MAPK10 (mitogen-activated protein kinase 10),* that is enriched for *Enzyme inhibitor activity/Response to radiation* was initially disconnected from the main network in the untrained rest state (see Fig. 7(a)). This module however was upregulated and ‘re-wired’ in the trained network state (see Fig. 7(b)), gaining several second-degree connections with *GABRAPL1*. Importantly, this module was also unique to the trained network (i.e. genes not associated with the exercise response) (see Fig. 7(c)). Arrestins such as ARRB1 are known to promote agonist-mediated desensitization of G protein-coupled receptors [129] and kappa and delta OR-mediated G protein activation has been shown to be attenuated by over-expression of ARRB1 in fibroblast-like cell lines [130]. This module is also extended with the addition of several genes also unique to the training response (see Fig. 7(c)), namely *CAT* (*catalase*) and *EGLN3* (*egl-9 family hypoxia-inducible factor 3*) and *JUN* (*Jun proto-oncogene, AP-1 transcription factor subunit*), which were also upregulated at a transcriptional level in the trained cohort. Catalase helps protect the cell against hydrogen peroxide mediated cytotoxicity especially when levels of pyruvate are low [131]. *EGLN3* is one of the most important isoenzymes in modulating the hypoxic response [132] and has also been reported to regulate myogenin expression and skeletal muscle differentiation. Interestingly, JUN is known to be activated by ORs [133] and has been found to be involved in oxidative stress-mediated suppression of insulin gene expression [134]. Another six-gene cluster in the training response network, that was upregulated in a coordinated manner in response to training, contained genes that encode ligands and receptors of the VEGF signalling pathway. Several of these, namely *KDR*, *NRP*1 and *FLT4*, were exclusive to the training response. In support of this, skeletal myofiber VEGFA has been found to be essential for the exercise training response in mice [135]. These data suggest that there may be coordinated upregulation of several genes specific to the training response that may control ancillary processes in the adaptive response to training in the skeletal muscle of the Thoroughbred.

## Conclusion

Functional category over-representation analysis is a commonly used and valuable aid to the biological interpretation of gene/protein lists. However, this analysis may be improved with additional information (e.g. molecular interactions inferred from gene expression) derived from the experimental context in question (e.g. post-exercise skeletal muscle) and a contribution from unsupervised methods (e.g. community analysis) in defining functional classes. The absence of these considerations risks underexploiting rich high-throughput datasets and overlooking novel information that might help to expand knowledge of biological systems. As this is the first demonstration of this computational method, we have applied a stringent implementation to maximize quality over scale; however, in the future more refined implementations could be developed. Our results clearly demonstrate that network analysis and its capacity to predict influential nodes is very useful for highlighting putative control points. For example, our hypothesis that *GABARAPL1* and autophagy may play a central role in the response to exercise and training was generated solely from this network analysis approach. Standard over-representation analysis enabled some insight into downstream effects (e.g. enrichment for energy metabolism, neurodegenerative disease etc.) but did not highlight the autophagy process nor point to a putative driver gene. Neither was this indicated by differential gene expression analysis (*GABARAPL1* was outside the top 250 genes most significantly associated with exercise or training). Therefore, we have demonstrated the benefits of a novel network-based approach, which may serve as a model for future transcriptomics-based investigations into the molecular dynamics of biological systems in health and disease.

Taken together our results, both from the over-representation analysis and our novel network analysis approach, support the involvement of mitochondrial respiratory complexes I (*NADH dehydrogenase*) and V (*ATP Synthase*) in both the exercise and training responses. As expected, the exercise response in the untrained cohort appeared to include elements of a stress response but also included the downregulation of genes involved in myogenic differentiation. Neither of these features were observed in the trained cohort, although we cannot preclude them from a ‘trained’ exercise response based on the current data. It is also noteworthy that there is some limited support for the constitutive downregulation of heatshock proteins in the trained cohort. However, we did observe strong indications of the constitutive and coordinated upregulation of the mitochondrial respiratory complexes I and V in the trained cohort, which may suggest that the skeletal muscle has adapted to regular bouts of intense-exercise in this manner, presumably improving the efficiency of oxidative phosphorylation. There is also the suggestion from our network analysis that this upregulation of mitochondrial complexes, and indeed mitochondria, in response to exercise and especially training may be controlled in part by autophagy (and perhaps mitophagy) via *GABARAPL1*. Our results also suggested a connection between *GABARAPL1* and the stimulation of/response to GPCR, especially via the opioid receptor, signalling, in both exercise and training. The network analysis also suggested that the *SPTBN1* gene may play an important role in the adaptive response and that this response may include re-wiring of its interactions at the post-synaptic neuromuscular junction. Somewhat unexpected was the strong overlap between the exercise and training responses and pathways linked to several neurodegenerative diseases. However, there seems to be a strong body of evidence for an established role for mitochondrial dysfunction and autophagy in neurodegenerative disease. This also supports our hypothesis of the central involvement of these processes in both the exercise and training response in skeletal muscle. The involvement of authophagy and mitochondrial activity at the synaptic junction in both neurodegenerative disease and athletic adaptation seems to be a common theme, but further validation of this largely in silico generated hypothesis is required. In recent years autophagy and its role in many cellular processes and diseases [136] has garnered much interest and already autophagy-enhancing therapeutics have been proposed [137]. This study has provided further support for this process in relation to exercise (especially intense exercise), provided novel evidence of its central involvement in the adaptation of skeletal muscle to training and identified the autophagosomal gene *GABARAPL1* as a potential regulatory target.

## Additional files

Additional file 1: Supplementary Information Main Document. (DOCX 110 kb)

Additional file 2: Supplementary Tables. (XLS 2823 kb)

## Abbreviations

AD: Alzheimer’s Disease
AMP: Adenosine Monophosphate
ATP: Adenosine Triphosphate
BP: Gene Ontology Biological Process
CC: Gene Ontology Cellular Component
DAVID: he Database for Annotation, Visualization and Integrated Discovery
GO: Gene Ontology
HD: Huntington’s Disease
KEGG: Kyoto Encyclopaedia of Genes and Genomes
KOR: kappa Opioid Receptor
MF: Gene Ontology Molecular Function
MHC: Myosin Heavy Chain
miRNA: micro-RNA
mRNA: messenger RNA
MSTN: Myostatin
MYOD/1: Myogenic differentiation 1
NADP: Nicotinamide adenine dinucleotide phosphate
OR: Opioid Receptor
PD: Parkinson’s Disease
PPI: Protein-protein interaction
PSICQUIC: Protein Standard Initiative Common Query Interface
TCA: The Citric Acid (Kreb’s) Cycle
TR: Trained rest cohort
UE: Untrained exercise cohort
UR: Untrained rest cohort
WD: Work Day
