# Supplementary material for: Equine skeletal muscle adaptations to exercise and training: evidence of differential regulation of autophagosomal and mitochondrial components
Source: BMC Genomics. 2017 Aug 9;18:595. doi: 10.1186/s12864-017-4007-9 (PMC5551008; doi:10.1186/s12864-017-4007-9)
Supplement: Supplementary file 1 — Supplementary Information Main Document. (DOCX 110 kb) [file 12864_2017_4007_MOESM1_ESM.docx]

**Equine skeletal muscle adaptations to exercise and training: evidence of autophagy-coordinated upregulation of mitochondrial components.**

Kenneth Bryan^1,*^, Beatrice A McGivney^1,*^, Gabriella Farries^1^, Paul A McGettigan^1^, Charlotte L McGivney^1^, Katie F Gough^1^, David E MacHugh^1,2^, Lisa M Katz^3^, Emmeline W Hill ^1,2, †^

^1^ UCD School of Agriculture and Food Science, University College Dublin, Belfield, D04 V1W8, Ireland.

^2^ UCD Conway Institute of Biomolecular and Biomedical Research, University College Dublin, Belfield, D04 V1W8, Ireland.

^3^ UCD School of Veterinary Medicine, University College Dublin, Belfield, D04 V1W8, Ireland.

^*^contributed equally to the research

^†^ Correspondence: emmeline.hill@ucd.ie

**Supplementary Information**

**1. Sequencing data details**

- 4 – 14 million reads per library
- 1.79 total reads
- 353GBases of sequence
- Median 88.4% unique alignment EquCab2
- Aligned to 26,991 Ensembl gene Ids

**2. Known Molecualar Interactions from the IntAct Database**

Known experimentally validated, Protein-Protein Interactions (PPIs) for human that were annotated to the IMEx (International Molecular Exchange consortium) standard [45] were downloaded from the *IntAct* database [46] using the PSICQUIC web service [47].

This dataset can be regenerated (in plain text tab delimeted format) from the following PSICQUIC query (placed in address bar of web browser):

*http://www.ebi.ac.uk/Tools/webservices/psicquic/intact/webservices/current/search/query/species:9606 AND annot:imex AND ptypeA:protein AND ptypeB:protein AND taxidA:9606 AND taxidB:9606 AND detmethod:experimental detection method AND negative:false?format=tab25*

*Note: to programmatically run this query (vua R’s download.file method for example) one must replace white spaces with the ‘%20’ special character.*

45. Orchard S, Kerrien S, Abbani S, Aranda B, Bhate J, Bidwell S, Bridge A, Briganti L, Brinkman FS, Cesareni G: **Protein interaction data curation: the International Molecular Exchange (IMEx) consortium**. *Nature methods* 2012, **9**(4):345-350.

46. Kerrien S, Aranda B, Breuza L, Bridge A, Broackes-Carter F, Chen C, Duesbury M, Dumousseau M, Feuermann M, Hinz U: **The IntAct molecular interaction database in 2012**. *Nucleic acids research* 2011:gkr1088.

47. del-Toro N, Dumousseau M, Orchard S, Jimenez RC, Galeota E, Launay G, Goll J, Breuer K, Ono K, Salwinski L: **A new reference implementation of the PSICQUIC web service**. *Nucleic acids research* 2013, **41**(W1):W601-W606.

**3.Top Hub GABARAPL1 Interactions:**

**3.1. Putative Exercise PPI Network:**

**-UTR (33):** ENSG00000107796, ENSG00000165637, ENSG00000169813, ENSG00000156709, ENSG00000117118, ENSG00000145362, ENSG00000091140, ENSG00000189043, ENSG00000158710, ENSG00000163346, ENSG00000163191, ENSG00000155363, ENSG00000117519, ENSG00000167815, ENSG00000132109, ENSG00000152234, ENSG00000115306, ENSG00000084754, ENSG00000136068, ENSG00000110955, ENSG00000111912, ENSG00000112118, ENSG00000136450, ENSG00000108561, ENSG00000073578, ENSG00000064601, ENSG00000075415, ENSG00000146701, ENSG00000126602, ENSG00000165629, ENSG00000124942, ENSG00000172757, ENSG00000239306

**-UTE (25):** ENSG00000107796, ENSG00000158710, ENSG00000163346, ENSG00000163191, ENSG00000155363, ENSG00000117519, ENSG00000132002, ENSG00000142453, ENSG00000138069, ENSG00000115306, ENSG00000184897, ENSG00000136068, ENSG00000111912, ENSG00000164938, ENSG00000178209, ENSG00000120694, ENSG00000187837, ENSG00000204390, ENSG00000169710, ENSG00000136450, ENSG00000101109, ENSG00000100600, ENSG00000166598, ENSG00000172757, ENSG00000081277

**-UTR *AND* UTE:**

ENSG00000107796, ENSG00000158710, ENSG00000163346, ENSG00000163191, ENSG00000155363, ENSG00000117519, ENSG00000115306, ENSG00000136068, ENSG00000111912, ENSG00000136450, ENSG00000172757

**3.2. Putative Training PPI Network:**

**-UTR (33):** ENSG00000165672, ENSG00000156709, ENSG00000117118, ENSG00000145362, ENSG00000091140, ENSG00000189043, ENSG00000167815, ENSG00000152234, ENSG00000115306, ENSG00000084754, ENSG00000110955, ENSG00000104823, ENSG00000108561, ENSG00000073578, ENSG00000101162, ENSG00000146701, ENSG00000126602, ENSG00000165629, ENSG00000121691

**-TR (25):** ENSG00000165672, ENSG00000156709, ENSG00000117118, ENSG00000117054, ENSG00000167815, ENSG00000152234, ENSG00000115306, ENSG00000084754, ENSG00000110955, ENSG00000104823, ENSG00000204390, ENSG00000128422, ENSG00000108561, ENSG00000073578, ENSG00000165629

**-UTR *AND* TR:**

ENSG00000165672, ENSG00000156709, ENSG00000117118, ENSG00000167815, ENSG00000152234, ENSG00000115306, ENSG00000084754, ENSG00000110955, ENSG00000104823, ENSG00000108561, ENSG00000073578, ENSG00000165629

**4. Overview of biopsy samples based on RNAseq (TPM)**

Principal Component Analysis (PC1 vs PC2) of Full Dataset


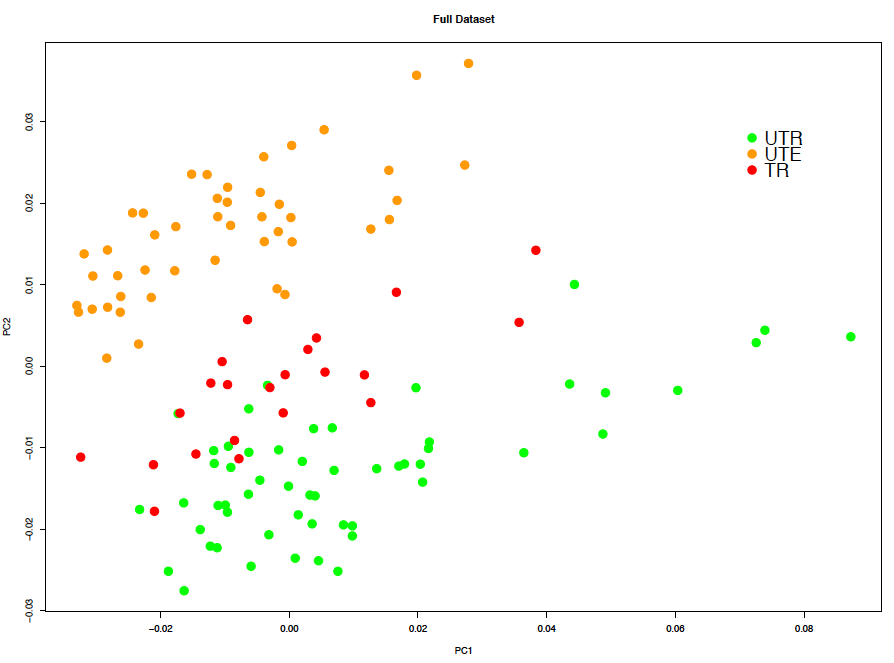


Principal Component Analysis (PC1 vs PC2) of Experimental Subset

**
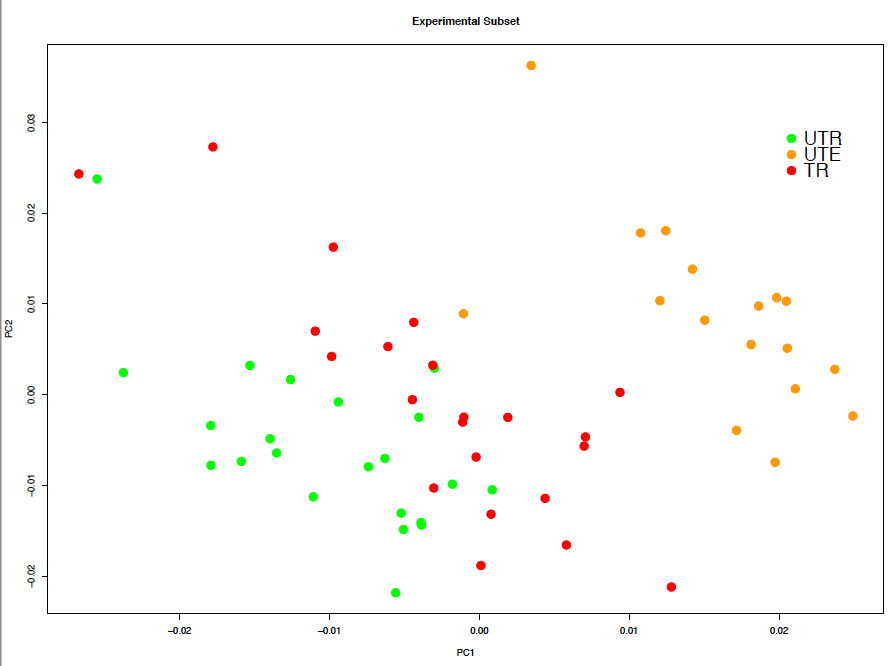
**

**5. Assessment of Age-related changes**

A small group of Untrained Rest (UR) were sampled after both training and a period of ‘detraining’ at rest (DR) ), i.e. after 2 weeks rest following their last exercise bout.

114 genes were found to be differentially expressed between DR and UR, this list is provided in Supplementary Table 7. No significant functional enrichment for KEGG or Reactome pathways or Gene Ontology terms was found within this list of genes. Of these 114 genes, 28 were also found to be differentially expressed between Untrained Rest (UR) and Trained Rest (TR) i.e. intersect with the genes in Supplementary Table 2.
